# Unveiling the Molecular Mechanism of Trastuzumab Resistance in SKBR3 and BT474 Cell Lines for HER2 Positive Breast Cancer

**DOI:** 10.3390/cimb46030171

**Published:** 2024-03-21

**Authors:** Anna Kokot, Sachin Gadakh, Indrajit Saha, Ewa Gajda, Michał Łaźniewski, Somnath Rakshit, Kaustav Sengupta, Ayatullah Faruk Mollah, Michał Denkiewicz, Katarzyna Górczak, Jürgen Claesen, Tomasz Burzykowski, Dariusz Plewczynski

**Affiliations:** 1 Department of Clinical Molecular Biology, Medical University of Bialystok, 15-089 Bialystok, Poland; rusek.annamaria@gmail.com (A.K.); e.gajda@gmail.com (E.G.); tomasz.burzykowski@uhasselt.be (T.B.); 2 Centre of New Technologies, University of Warsaw, 02-097 Warszawa, Poland; s.gadakh@cent.uw.edu.pl (S.G.); indrajit@nitttrkol.ac.in (I.S.); m.lazniewski@cent.uw.edu.pl (M.Ł.); somnath@utexas.edu (S.R.); kaustav.sengupta@pw.edu.pl (K.S.); afmollah@aliah.ac.in (A.F.M.); m.denkiewicz@cent.uw.edu.pl (M.D.); 3 Department of Computer Science and Engineering, National Institute of Technical Teachers’ Training and Research, Kolkata 700106, India; 4 Faculty of Mathematics and Information Science, Warsaw University of Technology, Koszykowa 75, 00-662 Warszawa, Poland; 5 Department of Mathematics and Statistics, Hasselt University, 3500 Hasselt, Belgium; katarzyna.gorczak@uhasselt.be; 6 Department of Epidemiology and Data Science, Amsterdam Universitair Medische Centra, VU University, 1081 HV Amsterdam, The Netherlands; j.claesen@amsterdamumc.nl

**Keywords:** microRNA, microarray, HER2, breast cancer, drug resistance

## Abstract

HER2-positive breast cancer is one of the most prevalent forms of cancer among women worldwide. Generally, the molecular characteristics of this breast cancer include activation of human epidermal growth factor receptor-2 (HER2) and hormone receptor activation. HER2-positive is associated with a higher death rate, which led to the development of a monoclonal antibody called trastuzumab, specifically targeting HER2. The success rate of HER2-positive breast cancer treatment has been increased; however, drug resistance remains a challenge. This fact motivated us to explore the underlying molecular mechanisms of trastuzumab resistance. For this purpose, a two-fold approach was taken by considering well-known breast cancer cell lines SKBR3 and BT474. In the first fold, trastuzumab treatment doses were optimized separately for both cell lines. This was done based on the proliferation rate of cells in response to a wide variety of medication dosages. Thereafter, each cell line was cultivated with a steady dosage of herceptin for several months. During this period, six time points were selected for further in vitro analysis, ranging from the untreated cell line at the beginning to a fully resistant cell line at the end of the experiment. In the second fold, nucleic acids were extracted for further high throughput-based microarray experiments of gene and microRNA expression. Such expression data were further analyzed in order to infer the molecular mechanisms involved in the underlying development of trastuzumab resistance. In the list of differentially expressed genes and miRNAs, multiple genes (e.g., *BIRC5*, *E2F1*, *TFRC*, and *USP1*) and miRNAs (e.g., hsa miR 574 3p, hsa miR 4530, and hsa miR 197 3p) responsible for trastuzumab resistance were found. Downstream analysis showed that *TFRC*, *E2F1*, and *USP1* were also targeted by hsa-miR-8485. Moreover, it indicated that miR-4701-5p was highly expressed as compared to *TFRC* in the SKBR3 cell line. These results unveil key genes and miRNAs as molecular regulators for trastuzumab resistance.

## 1. Introduction

Cancer continues to pose a substantial global health threat despite advancements in diagnosis and treatment [1]. In a recent update in 2020, there were an estimated 19.3 million new cases and 10.0 million cancer-related deaths [2], an increase from 18.1 million cases and 9.6 million deaths in 2018 [3]. This rise can be attributed to factors such as population growth, increased exposure to risk factors like smoking and obesity, and changing reproductive patterns due to economic development and urbanization. Lung cancer is the most frequently diagnosed and deadliest, followed closely by breast cancer. Breast cancer is the most common cancer in women worldwide, with over 2.3 million new cases in 2020, significantly contributing to cancer-related mortality [4]. Breast cancer is now recognized as a diverse group of diseases with distinct clinical behaviors, molecular components, risk factors, prognostic markers, and responses to treatment. Molecular classification relies on markers like estrogen receptors (ER), progesterone receptors (PR) and HER2 and Ki67 proliferation rate [5]. As a result, breast cancer has been categorized into five subtypes: luminal A, luminal B, triple-negative, and two HER2-positive types. Breast cancers with HER2 overexpression constitute 15–25% of cases, being aggressive and challenging to treat. Trastuzumab was approved by the FDA in 1998 and demonstrated a 37% relative improvement in overall survival with an increase of about 9% in the probability of 10-year OS when combined with chemotherapy [6]. Mutations in PIK3R1, activating PI3K/Akt/mTOR, drive trastuzumab resistance, especially in HER2-overexpressing breast cancer [7]. Approximately 70% of HER2-positive breast cancer patients develop resistance to trastuzumab within a year of treatment initiation, despite initial responsiveness [8].

In this context, the present study aimed to comprehensively investigate the molecular mechanisms underlying trastuzumab resistance using a two-fold approach. Initially, well-established breast cancer cell lines, namely SKBR3 and BT474 [9], were used to mimic the in vitro conditions representative of HER2-positive breast cancer. Optimal trastuzumab treatment doses were determined for each cell line based on their respective proliferation rates in response to a wide range of medication dosages. Subsequently, both cell lines were continuously cultivated with a steady dosage of trastuzumab over several months, leading to the development of trastuzumab-resistant cell lines. To capture the dynamic changes occurring during the acquisition of resistance, six distinct time points were selected for further analysis, encompassing the progression from the untreated cell line at the outset to the fully resistant cell line at the termination of the experiment. Concurrently, a second stage of the study involved the extraction of nucleic acids from the aforementioned cell lines for subsequent high-throughput microarray experiments targeting gene and microRNA expressions. The resulting expression data were subjected to comprehensive bioinformatics analyses to unravel the intricate molecular mechanisms underpinning the development of trastuzumab resistance. Through this approach, we focused on the 25 genes and microRNAs with the most statistically significant changes in expression levels across time, implicated in the development of trastuzumab resistance, and demonstrated crucial roles in protein–protein interactions. Notably, among the identified genes and microRNAs, *BIRC5*, *E2F1*, *TFRC*, and *USP1* emerged as the top candidates influencing trastuzumab resistance, while miR-574-3p, miR-4530, miR-8485, and miR-197-3p were identified as the key microRNAs regulating this process. In-depth investigations using the prediction by the miRDB database [10] highlighted the miR-4701-5p targeting *TFRC* and the targeting of *E2F1* and *USP1* by hsa-miR-8485. Moreover, the expression revealed substantial upregulation of miR-4701-5p compared to *TFRC* in the SKBR3 cell line, providing crucial insights into the intricate regulatory mechanisms governing trastuzumab resistance. Overall, the findings of this study shed light on the critical molecular players and pathways driving trastuzumab resistance in HER2-positive breast cancer, offering valuable insights into potential therapeutic targets and strategies for overcoming treatment challenges associated with this aggressive subtype of breast cancer.

## 2. Materials and Methods

### 2.1. Breast Cancer Cell Lines

SKBR3 and BT474 human breast cancer cell lines were chosen for this study due to their HER2 receptor overexpression, trastuzumab sensitivity [11,12,13], and potential for trastuzumab resistance [14,15]. These certified, mycoplasma-tested cell lines, obtained from American type cell collection, were used as controls and parental lines for drug-resistant cell line development. Despite sharing HER2 overexpression, their genetic backgrounds and origins differ.

### 2.2. Cell Culture Conditions

Cells were cultured in DMEM/F12 with 10% FBS and antibiotics to prevent contamination. Adherent cell cultures were maintained in T75 flasks at 37 °C with 5% CO_2_. Parental cell lines were cryo-preserved at 80–90% confluence with 10% DMSO/FBS. Fresh medium or trypsin-EDTA was used to maintain cell lines as needed.

### 2.3. Drug Resistance Development Conditions and Monitoring

**Figure 1:**
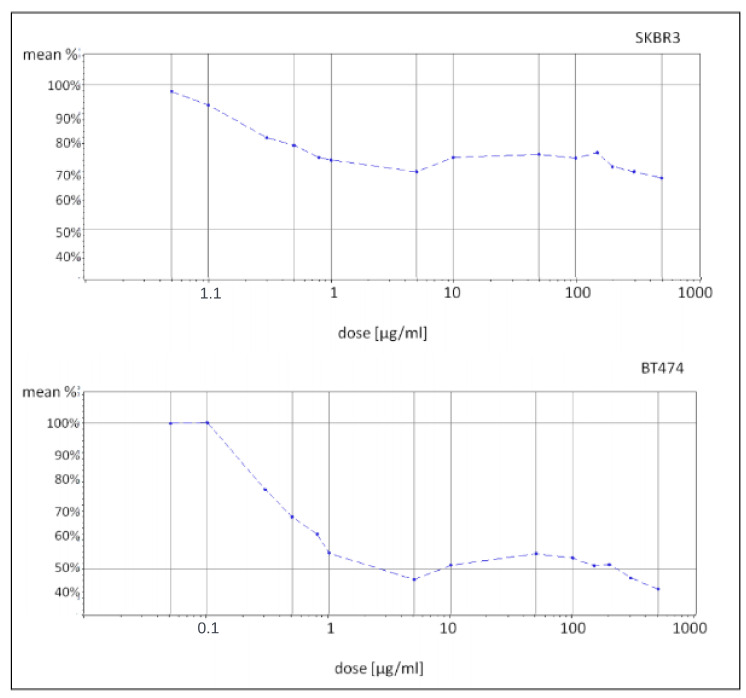
Plots representing the relationship between drug dose (“dose [μg/mL]”axis on the logorithmic scale) and proliferation intensity, presented as a percentage of the proliferation rate of drug-treated cells compared to the control untreated cells, based on proliferation assay measurements (“mean %” axis). Proliferation rates were measured based on six biological replicates and two technical replicates. The experiment was carried out in three independent repetitions.

A long-term, consistent dose of trastuzumab (Herceptin) was used to develop drug-resistant cell lines with a strong response. Initial experiments used a proliferation assay to test various drug doses (ranging from 0.05 μg/mL to 500 μg/mL) on two cell lines. As indicated in Figure 1, increasing the dose to more than 5 μg/mL and 10 μg/mL did not substantially decrease the proliferation rate. Consequently, doses of 5 μg/mL and 10 μg/mL were selected based on the aforementioned results and literature [16]. Control cells were cultured similarly without drug exposure. Regular proliferation assays were conducted, and cell samples were preserved for analysis. Over time, cells became more resistant, with SKBR3 cells starting to be more sensitive than BT474. Six key time points were chosen for further investigation, including a control.

### 2.4. RNA Extraction

Total RNA was extracted from frozen cell pellets using the MirVana TM Isolation Kit as per the manufacturer’s instructions, aiming to study mRNA and microRNA changes in the same material, as recommended by Agilent for microarray experiments.

### 2.5. Gene Expression Microarray Experiments

Gene expression microarray experiments utilized Agilent’s SurePrint G3 Human GE 8x60K v2 Microarrays, encompassing over 50,000 biological features, including long intergenic non-coding RNAs (linc RNAs). Labeled cRNA was generated from 200 ng of input, purified with the RNeasy Mini Kit, and quantified. Microarray slides were washed, scanned using an Agilent Scanner, and analyzed with Feature Extraction software.

### 2.6. MicroRNA Microarrays Experiments

MicroRNA expression experiments utilized Sure Print G3 Unrestricted miRNA 8x60K microarrays by Agilent Technologies, 5301 Stevens Creek Boulevard Santa Clara, CA, USA, designed based on the miRBase-microRNA database. One Color approach was employed with two replicates for each cell line and time point. RNA labeling and hybridization followed standard protocols, and microarray data were analyzed with Agilent Scanner and Feature Extraction software, all meeting quality control standards.

### 2.7. Gene and microRNA Expression Microarrays Data Analysis

Gene and microRNA expression data from BT474 and SKBR3 cell lines were separately analyzed. A total of 34,756 genes and 2549 microRNAs were examined, with some genes and microRNAs having multiple measurements. In particular, for each of the six time points, two separate replicates were placed on two (out of three) different slides in a balanced design to allow within-slide pairwise comparisons between the time points. Additionally, for some genes and microRNAs, there were multiple probes. Measurements resulting from all the replicates and probes were analyzed for each gene and microRNA. In particular, a linear model with slide and time-point factor (and a probe effect for genes with multiple probes) was used for genes. The model included slide, time point, and probe factors for microRNAs. The slide effect accounted for slide-to-slide variations and served as a normalization factor. ANOVA was used for genes with multiple measurements, and LIMMA [17] was employed for genes with non-replicated probes to enhance error variance estimation. In the analysis, the null hypothesis of no mean expression difference among all time points was tested first for both genes and microRNAs. The Benjamini–Hochberg procedure [18] was used to correct for multiple testing (FDR = 0.05). Upon rejection of the null hypothesis, Tukey’s multiple-testing procedure α = 0.05 was applied to conduct pairwise comparisons between time points.

### 2.8. Association of miRNA Target Genes

Based on the list of identified top 25 most significant genes and top 25 most significant miRNAs from both cell lines, we derived the miRNA target gene pairs using the miRDB database [10]. The miRDB database uses a Target score for target prediction, where the score is assigned by the miRNA target prediction program based on support vector machines (SVMs) and high-throughput training datasets. We then examined the relationship between miRNAs and their target genes by considering the distribution of log2FC scores for each pair.

### 2.9. Bioinformatics Analysis of Statistically Processed Microarray Data

Two initial gene datasets were generated by analyzing microarray data from BT474 and SKBR3 cell lines. Genes with *p*-values below 0.05 were chosen, resulting in 8874 genes for BT747 and 13,892 genes for SKBR3. A subsequent dataset was created by selecting 5674 genes that exhibited altered expression in both cell lines, meeting the same *p*-value threshold. To identify enriched Gene Ontology (GO) terms, the topGO package in R [19] was employed. It used a combination of “elim” and “weight” algorithms [20], considering the hierarchical structure of the GO database. The Fisher exact test assigned *p*-values for each GO term enrichment. Similarly, KEGG pathway enrichment analysis was conducted to identify pathways affected by trastuzumab treatment in BT747 and SKBR3 cell lines. Due to the complexity of KEGG pathways, the Fisher exact test was applied. Only genes analyzed with the SurePrint G3 Human GE 8x60K v2 Microarray were included. Additionally, protein–protein interaction analysis was performed by mapping gene names to Ensemble and Uniprot IDs, creating proteome networks based on various databases. The focus was on explaining the connections between the top 25 significant genes in each cell line via the proteomic network, seeking the shortest path within 17 hops. Eight sub-network images were generated for essential genes in both cell lines using the top four significant genes as reference points.

## 3. Results

In this section, we first describe the cancer cell response to trastuzumab, observing, notably, a temporal decline in the response. Six time points were selected within the studied time period for further microarray experiments. The microarray experiment results fulfill the acceptable quality control metrics for all the samples across different time points for RNA and miRNA. Next, we discuss the results of the microarray expression data, revealing statistically significant genes and miRNA, and their role in the development of trastuzumab resistance.

### 3.1. Identification of the Molecular Changes That Occur during the Emergence of Trastuzumab Resistance

#### 3.1.1. Cell Culture: Drug Resistance Development Conditions and Monitoring

Cell cultures were maintained in Dulbecco’s Modified Eagle Medium (DMEM) supplemented with F12 nutrient mixture, 10% fetal bovine serum (FBS), and antibiotics to prevent bacterial contamination. The cells were incubated in T75 treated flasks at 37 °C with 5% CO_2_. Before any passage, the parental cell lines were cultured without drug treatment until they reached 80–90% confluence and then cryo-preserved in 10% DMSO/FBS in liquid nitrogen. Additional vials were frozen as a backup at an early passage number. A prolonged and consistent dosage of trastuzumab (Herceptin, Roche, Engelhorngasse 3, Vienna, Austria) was administered to establish drug-resistant cell lines and determine an adequate response. Initial experiments evaluated the proliferation intensity following exposure to a wide range of drug doses (0.05 μ/mL–500 μg/mL) in both cell lines (Figure 1).

**Figure 2:**
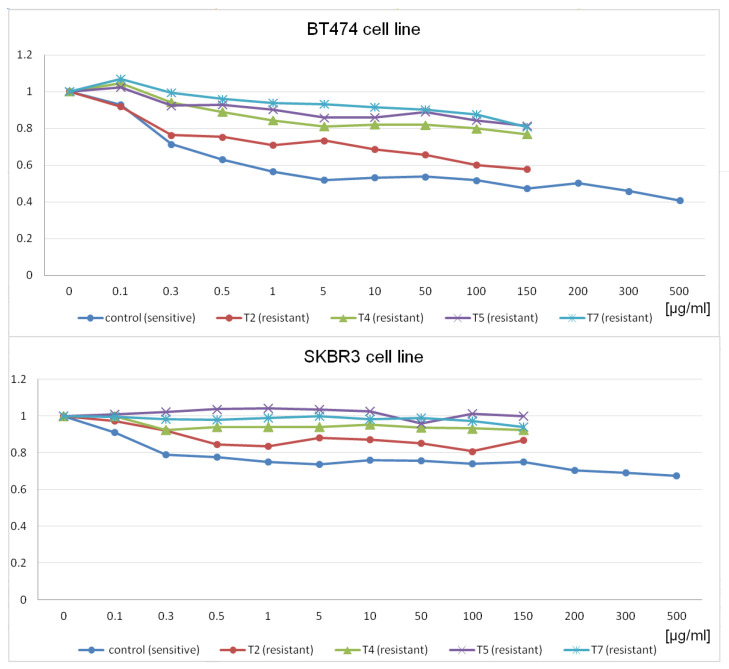
Diagrams presenting changes in proliferation rates at different time points of the experiment during the whole time of cell exposure to trastuzumab. The charts represent the relationship between drug dose (“[μg/mL]”axis) and proliferation intensity (y-axis) presented as a decimal fraction of the proliferation of drug-treated cells compared to the control untreated cells and are based upon proliferation assay measurements, with each curve corresponding to a particular time point. “Control” means cells purchased from ATCC and confirmed to be trastuzumab sensitive and able to develop resistance. Control cell lines were not treated with the drug at any time in the experiment and reaction for the drug was stable. T2, T4, T5, and T7 were treated at for 2, 4, 5, and 7 months, respectively. The time points were selected based on the significant increase in proliferation rates (decreased reaction for the drug) to the previous month, reflecting a gradual development of resistance to trastuzumab.

The cell response to Herceptin was assessed via a proliferation assay using the BioTek Cytation TM 3 imaging reader. Based on the preliminary findings and existing literature, two treatment doses (5 μg/mL and 10 μg/mL) were chosen. The control cell lines were cultured alongside the resistant cells, following the same procedures except for drug exposure. Regular proliferation assays were performed monthly to monitor the development of resistance by observing changes in cell response to the drug. At each time point, cell samples were preserved in liquid nitrogen or frozen at −80 °C for future analysis. At the commencement of the study, both the SKBR3 and BT474 cell lines showed differing levels of sensitivity to the drug, with the BT474 primary tumor cells displaying greater sensitivity compared to the metastatic SKBR3 cells, as evidenced by a 72% and 50% proliferation rate, respectively. Despite the difference, this indicates a successful establishment of sensitivity to trastuzumab. By the study’s conclusion, both cell lines exhibited increased proliferation intensity, with the SKBR3 cells reaching 92% and the BT474 cells reaching 89% as compared to untreated controls when exposed to a 100 μg/mL drug concentration. Furthermore, there was a discernible trend indicating a decrease in the cells’ responsiveness to the drug over time, suggestive of the development of resistance during the duration of exposure (Figure 2). Six specific time points, including the control, were selected for subsequent microarray experiments.

#### 3.1.2. RNA Extraction and Quality Evaluation

For each designated time point, total RNA was isolated from frozen cell pellets using the MirVana TM Isolation Kit (Ambion, Life Technologies, Carlsbad, CA, USA) as directed in the online protocol https://assets.fishersci.com/TFS-Assets/LSG/manuals/MAN0011131_A27828_magmax_mirvanatotalrna_manualextration_ug.pdf, accessed on 15 March 2024. This approach aimed to enable the examination of both mRNA and microRNA in the same material, in accordance with Agilent’s recommendations for microarray experimentation. Each RNA sample underwent rigorous assessment of its concentration and quality using the NanoDrop 2000 spectrophotometer (Thermo Scientific, Waltham, MA, USA) to ensure acceptable purity (260/280 nm: 1.9–2.1; 260/230 nm: 1.8–2.2). Additionally, Agilent Bioanalyzer was employed to evaluate concentration and integrity, ensuring a minimum RNA Integrity Number (RIN) of 7 for all samples (Figure S1). Repeated extractions and assessments were conducted for samples that failed the quality checks.

#### 3.1.3. Gene Expression Microarrays Experiment

The gene expression microarray analysis was conducted using the Sure Print G3 Human GE 8x60K v2 Microarrays from Agilent Technologies, designed to detect a wide array of biological features, including long intergenic non-coding RNAs (linc RNAs). Labeled cRNA targets were prepared from 200 ng of input using the One Color Low Input Quick Amp Labeling Kit and One-Color Spike-In Kit from Agilent Technologies, followed by purification with the RNeasy Mini Kit from Qiagen. The quality of the cyanine3CTP-labeled cRNA was assessed to ensure samples had an activity exceeding 6 pmol/μg, meeting the manufacturer’s standard for high-quality samples. Table 1 presents the results of labeled cRNA quality control. The hybridization procedure was performed overnight, with two replicates for each cell line at every time point. Microarray slides were washed using GE Wash Buffers and then scanned with an Agilent Scanner version C (G2505C). Feature Extraction software was utilized for image analysis, yielding raw data files, quality control PDFs for each array, and a comprehensive summary protocol. All arrays exhibited satisfactory quality control metrics, enabling a robust comparative analysis across different time points without the need for dye-swap experiments.

#### 3.1.4. MicroRNA Expression Microarray Experiment

The microRNA expression microarray experiment was conducted by utilizing the Sure Print G3 Unrestricted miRNA 8x60K microarrays (Agilent Technologies), encompassing probes for almost all known human microRNAs based on the miRBase database. Following the One Color approach, two replicates were performed for each cell line and time point. Starting from 100 ng of input, dephosphorylated and labeled RNA was prepared using the miRNA Complete Labeling and Hybridization Kit along with the microRNA Spike In Kit, followed by desalt procedures with the Micro Bio-Spin P6 Gel Column. The hybridization mixture, including Hyb Spike In, underwent overnight hybridization. Subsequently, microarray slides were washed and scanned in an Agilent Scanner version C (G2505C). Feature Extraction software was employed for image analysis (GRID: 070156DF20141006), confirming satisfactory quality control rates across all arrays.

### 3.2. Analysis of the Molecular Changes That Occur during the Emergence of Trastuzumab Resistance

#### 3.2.1. Gene Expression Analysis

**Figure 3:**
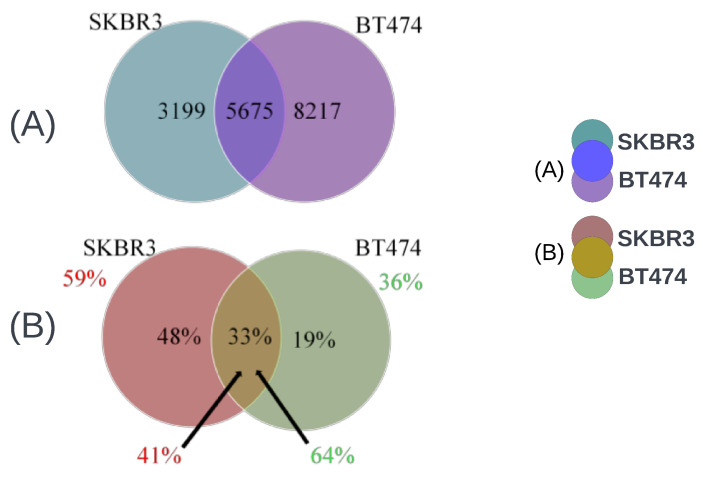
(**A**) Venn diagram shows the number of genes uniquely significant in SKBR3 and BT474 cell lines for trastuzumab resistance and the number of genes common to both. (**B**) Venn diagram to display the percentage of significant genes unique to SKBR3 and BT474 cell lines, the percentage of common significant genes, the percentage of genes significant in one cell line but important in the other (with an arrow), and the percentage of unique genes for each cell line among all significant genes in that cell line.

It is essential to note that only about one-third (5675/17,091 = 33%) of the genes with statistically significant expression-level changes in SKBR3 or BT474 were common to both cell lines. About 19% (3199/17,091) of the genes were unique to the SKBR3 cell line, while about 48% (8217/17,091) were specific to BT474. Furthermore, a substantial fraction (5675/8874 = 64%) of the genes important for BT474’s resistance development were also found to have statistically significantly altered expression levels in SKBR3, but the reverse was less true, with only 41% (5675/13,892) of genes related to SKBR3’s resistance development identified in BT474 (Figure 3).

**Figure 4:**
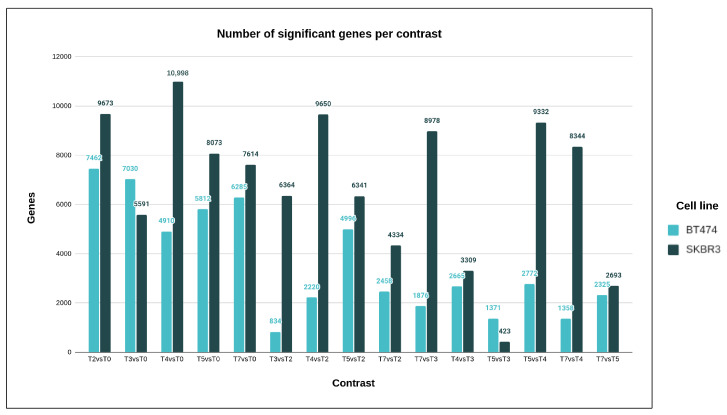
Bar chart presenting the number of genes (y-axis) with statistically significant differences between different pairs of time points (x-axis) for the two cell lines.

This disparity could be attributed to a significant imbalance in the number of significant transcripts found in both cell lines, with SKBR3 having more relevant genes identified as compared to BT474. In the second part of the statistical analysis, once the global null hypothesis of no change in expression levels across time was rejected for a gene, pairwise comparisons between all time points were conducted for that gene separately for each cell line (Table S1). The comparisons between T2 vs. T0 and T3 vs. T0 yielded the most statistically significant results, indicating that the most important global gene expression changes occurred at the beginning of trastuzumab treatment. This was followed by a temporary decrease in activity at T3, with more balanced gene expression modifications thereafter. Similar trends were observed in SKBR3 (Table S2), with the largest changes in gene expression occurring at the start of Herceptin exposure and a secondary increase in activity at T4. Pairwise comparisons are presented in (Figure 4).

In both cell lines, the majority of genes at each time point of drug resistance development were downregulated as compared to the parental cell line (Table 2), suggesting a potential role for tumor-suppressive genes in the process.

However, a different pattern emerged when considering sequential changes throughout the process. In BT474, 98% of genes were initially downregulated at the beginning of drug exposure (T2 vs. T0) (Table 3), followed by two instances of global gene overexpression (74% at T3 vs. T2 and 95% at T4 vs. T3) before more balanced changes toward the end of the process (60% vs. 40% in both T5 vs. T4 and T7 vs. T5). In SKBR3, while the global gene expression changes appeared more variable, they shared some similarities with BT474. The majority of genes (86%) were initially downregulated (T2 vs. T0) (Table 3), followed by a reverse situation (73% overexpressed at T3 vs. T2) and balanced expression patterns at the end (40% vs. 60% in T7 vs. T5) (Figure S2, Table 3).

These results suggest that trastuzumab had an inhibitory effect at the initial stages of the experiments, but as time progressed, cells seemed to adapt to the drug treatment, resulting in increasing global gene expression and dynamic changes throughout the process. A more detailed investigation focused on the most statistically important genes in the subsequent part of the gene expression profile analysis. The top 25 significant genes (Table 4, remaining significant genes are listed in Table S3) were identified for both BT474 and SKBR3 cell lines, and it was found that 80% of the top 25 genes in BT474 were among the top 200 in SKBR3. Conversely, as much as 92% of the genes were common. These findings underscore the consistency of the results and the substantial overlap between both cell lines, suggesting shared patterns in the development of drug resistance.

Research on the association between specific genes and the development of trastuzumab resistance in the BT474 cell line has yielded crucial insights. Among the 25 most significant genes identified (Table S4), CHAF1B stands out, with its steadily increasing expression during trastuzumab exposure, indicating a role in DNA replication and cell division, possibly as an effector. *E2F1*, a key transcription factor controlling cell cycle and apoptosis, appears linked to the molecular mechanism driving drug resistance. TRIT1, a mitochondrial tRNA modifier and potential tumor suppressor, shows expression changes suggesting complex regulation. ADM’s role, despite its distinct expression pattern, remains unclear due to its multifunctional nature. Additional candidates of interest include *BIRC5* and *USP1*, both prominent in both cell lines and potentially involved in apoptosis prevention. IGFBP3’s role in extending IGF’s half-life, a factor in HER2 signaling pathway crosslinking with trastuzumab resistance, is noteworthy. IGF2BP1’s mRNA binding and translational regulation may also play a role. *GSTM3* and *RASD1*, while having complex expression patterns, are associated with drug resistance. Genes like *KLK11*, *CENPE*, *TFRC*, and *KIAA0586*, though not fully understood, could be indirectly involved. Some genes with diverse expression profiles (*CEBPB*, *SNHG32*, *TNFRSF11B*) or unclear functions (*PMP22*, *PSEN1*, *PLAT*, *ZNF195*, *RND3*, and *ERO1L*) require further investigation into the context of drug resistance development. Table S5 presents the 25 genes with the most statistically significant differences associated with trastuzumab resistance in the SKBR3 cell line, each accompanied by a brief description of their molecular functions. However, it is worth noting that only eight of these genes (*CCL2*, *F8A1*, *PRMT6*, *DTL*, *RTN4IP1*, *RAB5IF*, *EXO1*, *FAHD1*) were among the top 25 genes with the most statistically significant differences, while the remaining 15 are listed in Table S17 (*PFDN6*, *LOXL2*, *MRPS23*, *HMOX1*, *WASHC5*, *PPIP5K2*, *AHSA1*, *DNAJA3*, *RAD50*, *SRRT*, *SUZ12*, *PSMD6*, *PCNA*, *TSFM*, *FAM25*). Several genes, such as *BIRC5*, *E2F1*, *TFCR*, *GSTP1*, *YWHAH*, *DTL*, *DOLK*, *NACC2*, *DDIT*, *BRACA1*, and *DNAJA3*, seem to play crucial roles in trastuzumab resistance development. *BIRC5* and *E2F1* were also significant in the BT474 cell line, suggesting a common mechanism. Furthermore, some genes are linked to the p53 pathway, a well-known tumor suppressor. For example, *NACC2* represses transcription and inhibits *MDM2*, stabilizing *TP53*. *DDIT4* is involved in p53-mediated apoptosis regulation and may connect to HER2 signaling through mTOR. *DNAJA3* interacts with both p53 and HER2 and stimulates Hsp70 chaperone activity. Other genes involved in DNA repair and damage response, like *BRCA2*, *PCNA*, and *RAD50*, are also implicated in trastuzumab resistance. While some genes have unclear associations, such as *CCL2* and *MGST2* related to immune response, they require further investigation. Additionally, Supplementary Materials (Supplementary Tables S3–S6) highlight the most statistically significant changes in gene expression during trastuzumab resistance development, including long non-coding RNAs and proteins with limited information, offering avenues for future research into the molecular mechanisms of resistance.

#### 3.2.2. MicroRNA Expression Analysis

The primary objective of the microRNA expression analysis was to identify microRNAs that played a statistically significant role at all stages of trastuzumab resistance development. Surprisingly, for nearly all tested microRNAs, the differences in expression levels over time were statistically significant, with 99.6% in BT474 and 99.3% in SKBR3 cell lines found to yield significant results at the significance level of 0.05 (adjusted for multiple testing). In contrast, gene expression analysis showed a more limited number of significant transcripts, only 25% in BT474 and about 40% in SKBR3 (Table 5). This disparity suggests that microRNA molecules play a substantial role in trastuzumab resistance development, highlighting their involvement in complex regulatory networks.

**Figure 5:**
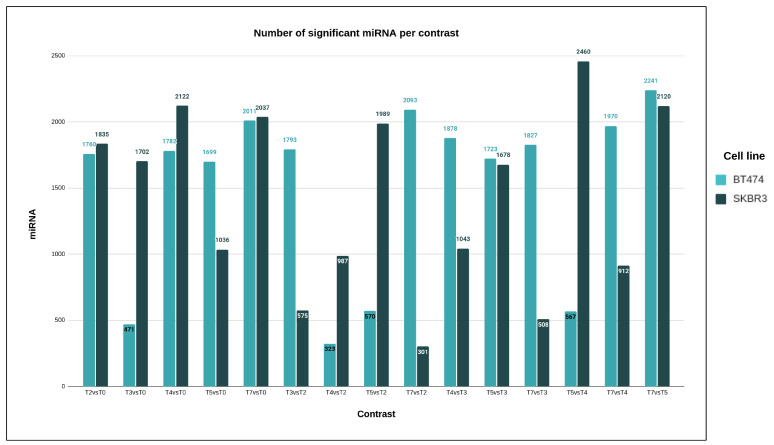
Bar chart presenting the number of microRNAs (y-axis) with statistically significant differences between different pairs of time points (x-axis) for the two cell lines.

Statistical analysis was used to examine microRNA expression changes in two cell lines, BT474 and SKBR3, during the development of drug resistance. Pairwise comparisons between different time points were conducted for each microRNA with a statistically significant test of the global null hypothesis of no change in expression across time (Figure 5). In the BT474 cell line, the T7 vs. T5 comparison showed statistically significant results for 88.2% of microRNAs, and 12.7% for T4 vs. T2. In the SKBR3 cell line, the T5 vs. T4, T4 vs. T0, and T7 vs. T5 comparisons yielded the highest significant results (96.8%, 83.5%, and 83.4%, respectively), while T7 vs. T2 yielded the lowest fraction of 11.8%. Both cell lines displayed a similar pattern of microRNA expression changes, but SKBR3 cells seemed to respond faster to changing conditions than BT474 cells (Table S6).

In the next phase of our microRNA expression profile analysis, we delved into the detailed study of the 25 microRNAs with the most statistically significant changes (Table 6, remaining significant miRNAs are in Table S8), focusing on their role in trastuzumab resistance within BT474 and SKBR3 cell lines. Surprisingly, 24 of the top 25 microRNAs in BT474 were also among the top 200 in SKBR3, suggesting substantial overlap in their resistance patterns. However, for the reverse scenario, we found less than 48% common microRNAs, indicating a stronger representation of BT474 results in SKBR3. Gene expression analysis further revealed that SKBR3 had a more complex network of genes and pathways involved in trastuzumab resistance, possibly affecting microRNA engagement and leading to this disparity in results.

Upon further analysis, we found that the eight most crucial microRNAs identified in the BT474 cell line were also among the top 50 (Table S8) microRNAs in the SKBR3 cell line. These microRNAs include hsa-miR-6886-3p, hsa-miR-4254, hsa-miR-4701-5p, hsa-miR-3151-3p, hsa-miR-6834-3p, hsa-miR-4281, hsa-miR-4649-3p, and hsa-miR-3162-3p. Additionally, two microRNAs with highly statistically significant changes discovered in the SKBR3 cell line, hsa-miR-6826-5p, and hsa-miR-6869-5p, were found within the top 50 (Table S8) important microRNAs in the BT474 cell line. These microRNA molecules may play a role in the development of trastuzumab resistance mechanisms. Four microRNAs, including hsa-miR-574-3p, hsa-miR-4530, hsa-miR-8485, and hsa-miR-197-3p, were identified among the 25 microRNAs with the most statistically significant results of the test of the global null hypothesis of no change in expression across time in both cell lines, suggesting their crucial role in trastuzumab resistance. Three of the four microRNAs (hsa-miR-574-3p, hsa-miR-4530, hsa-miR-8485) were among the 34 and 38 microRNAs (Table S7) in BT474 and SKBR3 cell lines, respectively, for which all pairwise timepoint comparisons were statistically significant. This underscores their potential importance in trastuzumab resistance development. Noteworthily, for hsa-miR-8485, all pairwise comparisons were significant in both cell lines.

#### 3.2.3. Analysis of miRNA Regulation of Genes

In our research, we employed a comprehensive approach by selecting the 25 genes and miRNAs with the most statistically significant changes from their respective cell lines. Our objective was to identify potential interactions between miRNAs and target genes using the miRDB database [10]. The miRDB predicted three pairs of miRNA-gene targets, as indicated in (Table 7).

**Figure 6:**
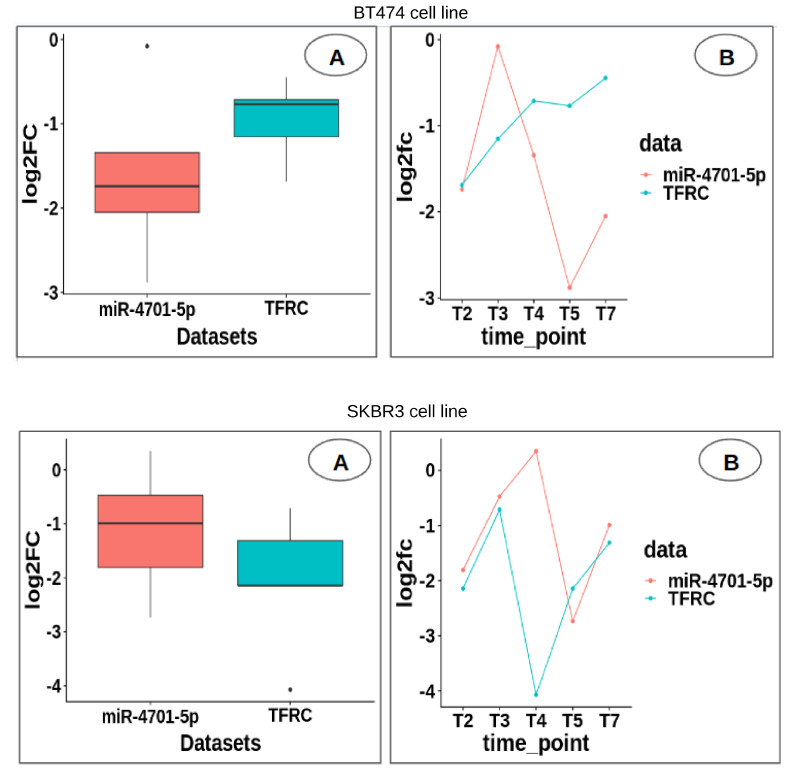
(**A**) Box plot and (**B**) line plot of the distribution of log2 fold change (log2FC) values of miRNA−target−gene pair, at their average and respective time points.

It is important to note that the miRDB predictions are assigned scores ranging from 50 to 100, with higher scores indicating greater statistical confidence in the prediction outcomes. Intriguingly, we observed that among these miRNA-gene pairs, four of the miRNAs and genes belonged to the top 25 genes and miRNAs with the most statistically significant changes identified in both cell lines. To gain deeper insights into the regulatory dynamics at play, we conducted an analysis of the association between miRNA-gene target pairs (miR-4701-5p-*TFRC* in Figure 6, figures for the remaining pairs from (Table 7) are presented in Supplementary Figure S17), and the distribution of log2 fold change (log2FC) values at their respective time points (Figure 6A) and average of time points (Figure 6B). This allowed us to investigate the extent to which miRNAs exerted control over gene expression within the context of the specific cell line under study.

#### 3.2.4. Gene Ontology Terms (GO Terms) Enrichment Analysis

This study leveraged high-throughput experiments to identify thousands of genes associated with trastuzumab resistance development in breast cancer. These genes exhibited diverse expression patterns throughout the study. The researchers conducted a Gene Ontology (GO) enrichment analysis [21] to gain insights into the underlying biological processes. The analysis focused on molecular function (MF) and biological process (BP) GO terms [22] separately for two cell lines, SKBR3 and BT474. SKBR3 showed 193 enriched MF GO terms (Table 8) and 600 enriched BP GO terms (Table 9), while BT474 had 103 (Table 10) and 354 (Table 11), respectively. Further investigation involved in-depth scrutiny of the top 20 statistically significant and the top 20 most overrepresented MF and BP GO terms for both cell lines. Gene significance density plots were generated to visualize gene distribution by *p*-value. To ensure result consistency within each cell line, the study examined the overlap between the most significant and most enriched GO terms. A comparative analysis between SKBR3 and BT474 included checking the overlap of GO terms, verifying enriched terms, and comparing gene significance density plots. Lastly, a global analysis was performed to categorize significant GO terms by molecular functions and biological processes, shedding light on the major functional groups driving trastuzumab resistance. Figures S3 and S4 depict density plots revealing the distribution of the top 20 statistically significant MF GO terms in the BT474 cell line. Five exhibits a symmetric distribution, encompassing broad actions like DNA binding and protein homodimerization. In contrast, most GO terms display a left-sided asymmetric distribution, including specific functions like DNA replication origin binding and mRNA 5′-UTR binding. Notably, a deeper analysis reveals that 10 of these terms are common between the most significant and enriched categories, emphasizing their importance in BT474. These findings highlight the crucial roles of these molecular functions in the studied process, as evidenced by their asymmetric gene significance enrichment profiles. Figures S5 and S6 display density plots of the top 20 most significant molecular function (MF) Gene Ontology (GO) terms in the SKBR3 cell line. Most SKBR3 MF GO terms exhibit symmetric distributions, while only nine show asymmetric distributions, with NADH dehydrogenase and single-stranded DNA binding being notable. Among these terms, nucleosomal DNA binding stands out as the most influential in the SKBR3 cell line despite some data inconsistency. Comparing the most significant Molecular Function Gene Ontology (GO) terms in two different cell lines, BT474 and SKBR3, revealed limited overlap, suggesting cell line-specific mechanisms in trastuzumab resistance. Three common GO terms were found, such as single-stranded DNA binding, RNA binding, and structural constituents of ribosome. However, their distribution patterns varied. SKBR3 displayed more asymmetric plots and a higher percentage of enriched GO terms, possibly due to its larger dataset. Despite differences, a substantial proportion of shared molecular functions implies the existence of some universal mechanisms contributing to trastuzumab resistance, albeit influenced by cell line-specific factors.

Figures S7 and S8 include density plots showcasing the top 20 most significant biological process (BP) Gene Ontology (GO) terms associated with the BT474 cell line. Like the molecular function (MF) GO terms, many BP GO terms exhibit asymmetric gene significance distributions. For example, “sister chromatid cohesion”, “DNA replication initiation”, and “SRP-dependent cotranslational protein targeting to the membrane” display left-skewed plots. In contrast, 8 out of the top 20 BP GO terms exhibit relatively symmetric distributions, including “cell division”, “cell proliferation”, and “DNA repair”. Interestingly, there is no direct correlation between the number of genes within a particular GO term and its gene significance distribution. However, there’s a tendency for these terms to occupy higher hierarchical positions. When comparing the most significant and enriched GO terms for BT474, only 4 out of 20 BP GO terms overlap. These common terms include “DNA replication initiation”, “regulation of transcription involved in G1/S transition of the mitotic cell cycle”, “NLS-bearing protein import into the nucleus”, and “positive regulation of pri-miRNA transcription by RNA polymerase II”. Furthermore, all these terms exhibit an asymmetric profile of gene significance enrichment, suggesting their importance in trastuzumab resistance in the BT474 cell line. Moving on to the SKBR3 cell line, Figures S9 and S10 depict density plots for the top 20 most significant BP GO terms. Unlike the MF GO terms, most BP GO terms in SKBR3 show asymmetric gene significance distributions, with left-skewed plots for terms such as “mitochondrial translational elongation” and “mitotic cytokinesis”. Conversely, 8 out of 20 BP GO terms display relatively symmetric gene significance distributions, including “cell division” and “DNA repair”. Unlike the BT474 cell line, there is no clear correlation between gene significance distribution, the number of genes in a GO term, or its hierarchical level in SKBR3. Notably, there is no overlap between the most significant and most enriched GO terms for SKBR3, indicating the complexity of processes involved in trastuzumab resistance in this cell line. Comparing the most significant BP GO terms between SKBR3 and BT474, 7 out of 20 are shared, such as “cell division”, “DNA repair”, and “regulation of signal transduction by p53 class mediator”. However, the gene significance density plots reveal diversity, with some terms showing asymmetric distributions in one cell line and symmetric distributions in the other. This underscores the importance of certain biological processes, like “G1/S transition of mitotic cell cycle”, in drug resistance development. In summary, while commonalities exist in the main processes contributing to trastuzumab resistance between SKBR3 and BT474, specific aspects and regulatory components differ, highlighting the cell line-specific nature of these mechanisms. Nevertheless, universal biological processes are shared across both cell lines, emphasizing their significance in trastuzumab resistance.

In the final phase of the Gene Ontology study, significant GO terms were analyzed separately for the BT474 and SKBR3 cell lines to uncover common molecular and cellular factors driving resistance to trastuzumab. The analysis revealed that critical molecular functions related to drug resistance included receptor binding (such as estrogen, retinoic acid, and death receptors), protein kinase activities, GTPase-related functions, and ATP/ATPase mechanisms. Additionally, significant functions were linked to transferase activities, RNA processing, DNA replication and repair, and p53 binding. Key biological processes involved cell cycle regulation, mitochondrial function, apoptosis, microRNA activity, stress response, viral infection, microtubule organization, and DNA damage repair. Several pathways, including Wnt signaling and insulin receptor pathways, were also affected during trastuzumab treatment and resistance development. For a detailed list of GO terms, refer to the Supplementary Materials.

#### 3.2.5. KEGG Pathways Enrichment Analysis

Section 2.1 highlights the complexity of trastuzumab resistance, involving numerous genetic factors. To gain deeper insights into the underlying biological processes and functional interpretation of high-throughput data, Section 3.2.4 focuses on Gene Ontology (GO) enrichment analysis. This method helps identify significant molecular functions and biological processes driving trastuzumab resistance, shedding light on the primary mechanisms at play. Additionally, the section delves into KEGG Pathways enrichment analysis, initiated by Professor Minoru Kanehisa in 1995 as part of the Japanese Human Genome Program [23]. KEGG Pathways database offers manually curated pathway maps encompassing molecular interactions, reactions, and networks involving genes, proteins, RNAs, chemical compounds, and more. These pathways span various categories, including replication, metabolism, transcription, and cellular processes, providing a comprehensive understanding of biological processes [24]. This analysis examines genes identified as significant in trastuzumab resistance development in BT474 and SKBR3 cell lines. Notably, the study reveals that Herceptin treatment significantly affects 9 pathways in BT474 and a striking 75 pathways in SKBR3. This discrepancy aligns with findings from the GO term analysis and gene expression studies, underscoring the complexity of trastuzumab resistance. The complete list of significant KEGG Pathways for both cell lines can be found in the Supplementary Materials. A comprehensive comparative analysis was conducted on the significance of various pathways in the BT474 and SKBR3 cell lines (Table S13 and Table S14, respectively), focusing on their response to trastuzumab treatment. Three common key pathways were identified, cell cycle, cellular senescence, and DNA replication, signifying their importance in both cell lines. Examining overrepresented pathways (Table S15 and Table S16, respectively), SKBR3 displayed a notably higher percentage of genes significantly affected within KEGG pathways (ranging from 76% to 94%) compared to BT474 (ranging from 43% to 65%).

Four pathways—DNA replication, cell cycle, colorectal cancer, and bladder cancer—were prominent in both cell lines, highlighting their significance. Furthermore, in the SKBR3 cell line, the 20 most significant and 20 most overrepresented KEGG pathways shared seven pathways related to molecular activities (e.g., DNA replication, cell cycle, apoptosis) and tumor development (e.g., pancreatic cancer, colorectal cancer). Interestingly, eight pathways (Table 12) were found to be involved in drug resistance development in both cell lines, underscoring the substantial overlap between these biological cell lines. Notably, the SKBR3 cell line revealed greater complexity in molecular activities induced by Herceptin exposure compared to BT474. These findings emphasize common resistance mechanisms while acknowledging the unique aspects of each cell line’s response to treatment.

#### 3.2.6. PPI Network Analysis

**Figure 7:**
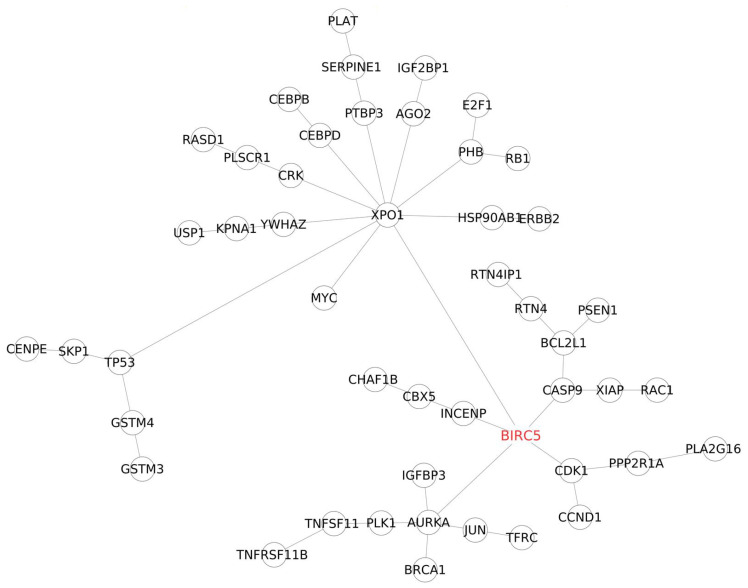
Protein–protein interaction (PPI) sub-network focused on the BIRC5 gene, incorporating the top 25 most significant genes in the BT474 cell line and genes associated with trastuzumab resistance development.

**Figure 8:**
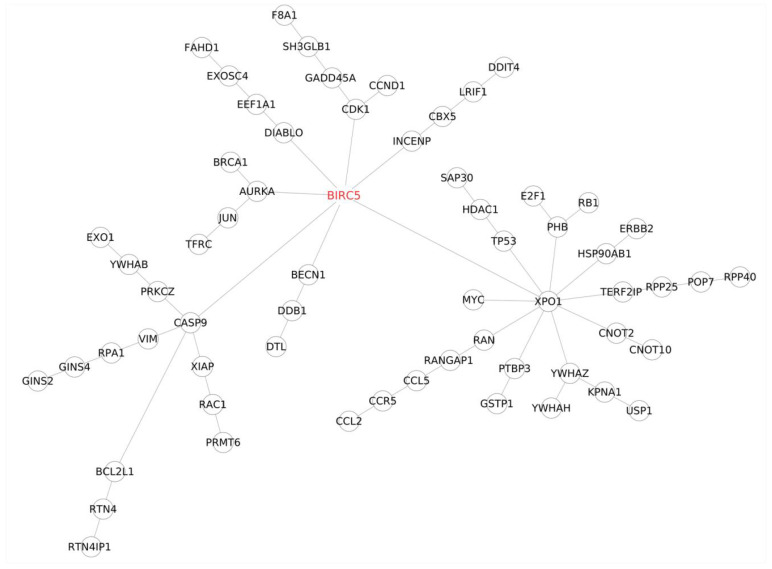
Protein–protein interaction (PPI) sub-network focused on the BIRC5 gene, incorporating the top 25 most significant genes in the SKBR3 cell line and genes associated with trastuzumab resistance development.

Integrating various “omics” data is essential for understanding intricate cellular-level biological processes. In this study, a straightforward ID mapping method was employed to link genomic data to proteomic data, focusing on protein-coding genes in the complete human genome and proteome. To navigate complex biological interactions, the study favored protein–protein interaction (PPI) networks over genomic networks due to their higher density and connectivity. Unlike gene–gene interaction networks, PPI networks provided a more reliable means to identify the shortest path between essential genes associated with trastuzumab resistance. The statistical analysis of the PPI network, particularly the Gold set of interactions, is summarized in (Table 13). The study then delved into detailed PPI network analyses for the top 25 significant genes in BT474 and SKBR3 cell lines, complemented by literature-reported trastuzumab resistance-related genes. The results revealed a close and complex interplay among these significant genes, i.e., *BIRC5* (Figure 7), (Figure 8), *E2F1* (Figures S11 and S12), *USP1* (Figures S15 and S16), and TFRC (Figures S13 and S14), shedding new light on the pathways and networks involved in trastuzumab resistance development.

Remarkably, *BIRC5*, *E2F1*, and *RB1* are pivotal players at the core of these networks, engaging with numerous proteins. As depicted in Figure 7 and Figure 8, *BIRC5* forms five direct connections shared across both cell lines: caspase 9 (*CASP9*), exportin (*XPO1*), Aurora kinase (*AURKA*), cyclin-dependent kinase 1 (CDK1), and inner centromere protein (INCENP) [25]. *CASP9*, linked to apoptosis, *AURKA*, involved in cell cycle regulation and tumorigenesis, and CDK1, essential for cell cycle progression, are vital contributors. *XPO1* stands out due to its multiple connections and its role in regulating protein transport. In SKBR3 cells, *BIRC5* also connects directly with *DIABLO* and *BECN1*, influencing apoptosis and autophagy [14]. Additionally, *BIRC5* interacts indirectly with *BRCA1*, *TFRC*, *IGFBP3* via *AURKA*, and *RAC1* via *CASP9*, forming numerous connections via *XPO1*, including *MYC*, *RB1*, *E2F1*, and *IGF2BP1* [14].

## 4. Discussion

The primary aim of this research was to delve into the molecular mechanisms responsible for developing resistance to trastuzumab, a drug used in breast cancer treatment. Traditional studies on Herceptin resistance mainly focused on identifying differences between resistant and sensitive cells [26,27,28]. However, this approach has limitations because cells can actively adapt to changing environments and treatment conditions. To address this, we proposed a novel approach combining a longitudinal investigation of in vitro resistance development with high-throughput microarray technology. Our comprehensive analysis of gene expression data encompassed over 34,000 genes and identified 8873 and 13,891 genes significantly contributing to trastuzumab resistance in two breast cancer cell lines, BT474 and SKBR3, with 5675 genes common to both cell lines. Further analysis grouped these genes into various molecular functions and biological processes using Gene Ontology terms. Additionally, we identified significant signaling pathways through KEGG Pathway analysis, with eight pathways commonly affected in both cell lines. Our study highlights the complexity of trastuzumab resistance development, emphasizing the need for a multi-dimensional understanding of the process. This research contributes valuable insights into potential targets for more effective breast cancer treatments [29,30]. In both cell lines, four genes—*BIRC5*, *E2F1*, *USP1*, and *TFRC*—emerged as highly significant transcripts within the top 25. *BIRC5* and *E2F1* exhibited a distinct expression pattern throughout the study. They initially decreased upon drug exposure and then gradually increased, mirroring the gradual development of drug resistance. This pattern aligns with existing research on *BIRC5*’s role in trastuzumab resistance, where the overactive PI3K/Akt [31] pathway leads to survivin overexpression, contributing to resistance [32]. In patients with HER2-overexpressing breast cancer, higher pretreatment survivin RNA levels correlated with poorer responses to trastuzumab, indicating *BIRC5*’s involvement in primary Herceptin resistance [32]. *E2F1*, a member of the E2F transcription factor family, shares similarities with survivin (BIRC5) in contributing to trastuzumab resistance. *E2F1* regulates cell growth and apoptosis, often activated in response to DNA damage [33]. Fanconi anemia DNA repair pathway emerged as crucial in trastuzumab resistance through KEGG pathway analysis. *E2F1* can also activate *BIRC5*, a known resistance factor. *E2F1* is linked to HER2 signaling and trastuzumab actions, with research showing its involvement in proliferative breast tumors. Interestingly, *E2F1* expression levels rise during trastuzumab exposure, indicating its potential role in drug resistance through independent HER2 pathway activation. While the roles of *BIRC5* and *E2F1* in drug resistance in HER2 breast cancer are understood, the involvement of *USP1* and *TRFC* remains unclear. *USP1* has been linked to cancer development and metastasis but not yet to trastuzumab resistance. Interestingly, *USP1* inhibitors have shown promise in leukemia treatment. Suppressing *USP1* leads to the degradation of the ID1 transcription factor, crucial for cancer progression. The high representation of *USP1* in molecular function and biological process gene ontology terms suggests its significant role in trastuzumab resistance mechanisms. Unlike *USP1*, current scientific literature does not mention *TFRC*’s role in Herceptin resistance development. Recent research has revealed *TFRC*’s involvement in various cancer-related signaling pathways, notably the endocytosis pathway, which is significant in trastuzumab resistance. While *TFRC*’s contribution to Gene Ontology terms is less pronounced than *USP1*, it plays a role in the “response to drug” biological process. This study emphasizes the importance of further investigating both genes in trastuzumab resistance development, as their correlation with drug resistance is not yet clear, and their molecular functions in the context of cancer progression and trastuzumab resistance require deeper exploration.

Understanding the molecular mechanisms behind resistance to Herceptin is critical. This study identified several genes, such as *BIRC5*, *BRCA1*, *RB1*, *ERBB2*, and others, as significant players in trastuzumab resistance, supporting previous research. New candidate genes like *E2F1*, *USP1*, and *TFRC* were also highlighted. Some genes, like *IGF2BP1* and *GSTP1*, showed potential involvement but require further confirmation. Surprisingly, this study did not confirm the involvement of certain previously reported genes in resistance. The findings suggest that primary Herceptin resistance may be linked to alterations in downstream components of HER2 signaling pathways or antiapoptotic proteins, rather than HER2 receptor activity itself. In contrast, acquired resistance may involve changes at the receptor level, such as epitope masking or upregulation of receptor components. Some intrinsic resistance mechanisms may overlap with acquired resistance, aligning with earlier research. Overall, this study underscores the complexity of trastuzumab resistance involving both HER2-dependent and independent pathways. Our study significantly advances our understanding of trastuzumab resistance by identifying new molecular contributors and genetic pathways. This knowledge informs future cancer genetics and molecular medicine research, potentially leading to effective adjuvant therapies. However, clinical validation is crucial, as preclinical and in vitro findings may not always translate. Additionally, we uncovered unexplored long non-coding RNAs and proteins, offering opportunities for further basic research.

This study unequivocally demonstrates the substantial role of microRNA molecules in developing resistance to trastuzumab. In both cell lines, more than 99.6% of the tested human microRNAs exhibited statistically significant expression changes during the development of drug resistance, even when applying stringent statistical criteria. Notably, using a more rigorous threshold for medical purposes revealed a high percentage of significant microRNAs, emphasizing the specificity of microRNA action. MicroRNA molecules play crucial roles in cellular processes such as proliferation, development, metabolism, differentiation, and apoptosis [34,35]. Their ability to target multiple mRNAs due to imperfect matching and their widespread regulatory influence on protein-coding genes further highlight their complexity [34]. Despite constituting only a small portion of the human genome, microRNAs are predicted to regulate a substantial portion of protein-coding genes. Four microRNAs, specifically hsa-miR-574-3p, hsa-miR-4530, hsa-miR-8485, and hsa-miR-197-3p, ranked among the top 25 most significant microRNAs in both cell lines, underscoring their significant involvement in the development of trastuzumab resistance. In silico analysis was conducted to identify potential targets of specific microRNAs (miRNAs) associated with Herceptin resistance in breast cancer cell lines. For hsa-miR-574-3p, TargetScan predicted potential targets such as *RAC1*, *BIRC5*, *E2F1*, *PMP22*, and *EGFR* [36]. Luciferase reporter assays confirmed direct regulation of *RAC1* and *EGFR* by miR-574-3p, both implicated in Herceptin resistance [37]. Similarly, miR-4530 showed potential targets, including FOXO1, MAPK4, and AKT predicted by miRDB [10] whereas Target Scan predicted *BIRC5*, *TFRC*, *HER2*, *ESR2*, and AURKA [36], some of which are involved in HER2 signaling and were important in Herceptin resistance. Another miRNA, hsa-miR-8485, was found to potentially target genes like *BIRC5*, *E2F1*, *USP1*, *RAC1*, *EPHA2*, *PTEN*, and *CCND1* [10,36], where, *E2F1* and *USP1*, being from the top four, were highly significant in both cell lines. Additionally, miR-4701-5p was identified as targeting TFRC [10], which was highly significant in both cell lines. These findings provide insights into the molecular mechanisms underlying Herceptin resistance in breast cancer. The Hsa-miR-197-3p, a microRNA, plays a crucial role in cancer progression, particularly in breast, bladder, and thyroid cancers. Multiple studies emphasize the influence of long non-coding RNAs (ncRNAs) on regulating miR-197-3p expression. LIFR-AS1 inhibits cell proliferation, migration, and invasion in breast cancer by repressing miR-197 [38]. Similar results were observed in bladder [39] and thyroid cancers [40], where miR-197-3p downregulation led to decreased cell proliferation, migration, and invasion due to the actions of specific ncRNAs. Notably, miR-197-3p is linked to the PTEN/PI3K-Akt pathway [40], which plays a key role in HER2 signaling. This suggests that miR-197-3p might be involved in an alternative pathway compensating for trastuzumab’s therapeutic effects, a drug targeting HER2. Additionally, miR-197 targets *MAPK1*, a gene associated with trastuzumab resistance. Overexpressing miR-197 can reverse drug resistance by inhibiting *MAPK1*, as seen in gastric cancer cells [41]. Overall, miR-197-3p has a significant role in trastuzumab resistance development, potentially through its regulation of *MAPK1* and involvement in alternative signaling pathways [41]. Furthermore, miR-197-3p directly regulates other genes implicated in trastuzumab resistance, including *FOXJ2* [42], *MTHFD1* [43], *RAN* [44], *TUSC2* [45], and *FUS1* [46], though their specific roles in drug resistance and cancer progression require further investigation. These findings support the hypothesis that miR-197-3p is a key player in developing resistance to trastuzumab, a critical drug in breast cancer treatment.

In summary, our study suggests that nearly all known human microRNAs may play a role in developing resistance to the drug trastuzumab. We identified four microRNAs that are particularly important in both biological cell lines studied. Two of these microRNAs are confirmed to be involved in either HER2 signaling or drug resistance, supporting our findings’ reliability. The other two highly significant microRNAs, which have limited existing information, were identified through computational analysis as potential regulators of genes associated with trastuzumab resistance. However, further research is needed to validate these hypotheses and understand the underlying mechanisms.

## 5. Conclusions

The current study conducted high-throughput microarray experiments to investigate the intricate dynamics of gene and microRNA expression changes during the development of trastuzumab resistance in two prominent breast cancer cell lines, BT474 and SKBR3. The analysis of a pool of 34,000 genes revealed distinct patterns of differential expression, with 8874 and 13,892 genes implicated in resistance development in BT474 and SKBR3, respectively. Remarkably, our findings highlighted the significant involvement of key genes, including *BIRC5*, *E2F1*, *USP1*, and *TFRC*, which are crucial players in both cell lines, particularly within the context of HER2 signaling. Moreover, the identification of novel contributors to Herceptin resistance, such as *IGF2BP1*, *GSTM3*, *RASD1*, *KLK11*, *GSTP1*, *YWHAH*, *DTL*, *DOLK*, *NACC2*, *DDIT*, and *DNAJA3*, among the top 25 significant genes, suggests the existence of previously unrecognized mechanisms in drug resistance. Importantly, our research also underscored the role of established genes, including *BIRC5*, *E2F1*, *BRCA1*, *RB1*, *ERBB2*, *EPHA2*, *IGFBP3*, *ADAM10*, *FOXM1*, *RAC1*, *MYC*, *CCND1*, *PTEN*, *TP53*, *MAP2K4*, and *PI3KCA* in contributing to resistance. Notably, protein–protein interaction analysis illuminated the pivotal roles of *BIRC5*, *E2F1*, and *RB1* as central hubs within networks linked to Herceptin resistance. Furthermore, the significant impact of long non-coding RNAs and microRNAs in this resistance context was evident, indicating their potential as vital regulators in the process. Gene Ontology analysis highlighted enriched molecular functions such as receptor binding, protein kinase activities, and DNA replication, while biological processes encompassed crucial aspects like cell cycle regulation, apoptosis, and DNA damage repair. Pathway analysis brought to light 9 and 75 affected networks in BT474 and SKBR3, respectively, with the convergence of eight common pathways, notably including cell cycle and p53 signaling. Notably, our investigation revealed HER2-dependent and independent resistance mechanisms, thereby ruling out the involvement of epitope masking and other ERBB receptors. Noteworthy complexities observed in SKBR3 possibly arose from disparities in the cancer stage, considering the primary vs. metastatic distinction. Intriguingly, our study highlighted the significant role of microRNAs in Herceptin resistance, with hsa-miR-574-3p, hsa-miR-4530, hsa-miR-8485, and hsa-miR-197-3p emerging as critical contributors, including some previously unreported microRNAs specific to each cell line. These comprehensive findings shed light on the multifaceted landscape of trastuzumab resistance in breast cancer, providing valuable insights for the development of more effective therapeutic strategies and personalized treatment approaches. Admittedly, the findings have been obtained based on an analysis of a limited amount of material (that included, due to resource constraints, only two independent cell-line replicates at each time point). Evaluation of their credibility should take this aspect of the study into account. In this respect, further validation of the findings would be important. For instance, an in vivo validation and analysis of gene expression at the protein level could be used to ensure the accuracy and comprehensiveness of the microarray-based findings. An investigation of the expression of identified DEGs in samples of patients included in clinical databases and of the association with patients’ outcomes could shed light on the relevance of the study’s findings to clinical practice. These extensions are left for future research. 

## Figures and Tables

**Table 1:** The results of labeled cRNA quality control. R—repeated extraction; the number of “R” means the number of extraction repetitions.

Sample ID	RNA ng/μL	Yield μg	260/280	DyeConc. ng/μL	Activity pmol/μg
BT474 controlRR	351.3	10.539	2.19	7.5	21.34927412
SKBR3 controlRRR	426.7	12.801	2.18	8.7	20.38903211
SKBR3 T2	221.4	6.642	2.23	2.6	11.74345077
BT474 T2	269.2	8.076	2.25	3.5	13.00148588
SKBR3T5C	241.4	7.242	2.21	3.2	13.25600663
BT474 T5	378.6	11.358	2.22	6.8	17.96090861
SKBR3 T7	333.3	9.999	2.23	4.9	14.70147015
BT474 T7R	354.5	10.635	2.21	5.8	16.36107193
SKBR3T3R	374.4	11.232	2.2	5.6	14.95726496
BT474 T3RR	387.3	11.619	2.19	6.2	16.00826233
SKBR3T4R	311.6	9.348	2.2	5.2	16.68806162
BT474 T4R	443.5	13.305	2.2	8.6	19.39120631

**Table 2:** Number of genes for which statistically significant changes of expression levels across time were obtained and which were either up-regulated or down-regulated.

	BT474				SKBR3			
Time-Point	Up	% of Signif	Down	% of Signif	Up	% of Signif	Down	% of Signif
T2 vs. T0	174	2	7288	98	1328	14	8345	86
T3 vs. T2	614	74	220	26	4670	73	1694	27
T4 vs. T3	1790	95	86	5	648	7	8330	93
T5 vs. T4	892	32	1881	68	7788	83	1544	17
T7 vs. T5	1389	60	936	40	1645	61	1048	39

**Table 3:** Number of genes for which statistically significant changes of expression levels across time were obtained and which were either up-regulated or down-regulated at a specific time point when compared with the control (parental) cell line.

	BT474				SKBR3			
Time-Point	Up	% of Signif	Down	% of Signif	Up	% of Signif	Down	% of Signif
T2 vs. T0	174	2	7288	98	1328	14	8345	86
T3 vs. T0	267	4	6763	96	500	9	5091	91
T4 vs. T0	342	7	4568	93	402	4	10,596	96
T5 vs. T0	562	10	5250	90	550	7	7523	93
T7 vs. T0	502	8	5783	92	341	4	7273	96

**Table 4:** List of the 25 genes with the most statistically significant changes during the whole period of trastuzumab resistance development in both cell lines: BT474 and SKBR3.

**Top 25 Most Significant in BT474 Cell Line**			
**Gene**	**Adj** ***p*****-Val**	**Gene**	**Adj** ***p*****-Val**
*KLK11*	7.6 × $10^{103}$	*ZNF195*	2.0 × $10^{-51}$
*IGF2BP1*	3.4 × $10^{-72}$	*C6orf48*	2.2 × $10^{-51}$
*GSTM3*	1.6 × $10^{-65}$	*ADM*	2.7 × $10^{-51}$
*IGFBP3*	3.5 × $10^{-64}$	*RND3*	2.7 × $10^{-51}$
*TNFRSF11B*	1.1 × $10^{-63}$	*CBPB*	1.9 × $10^{-50}$
*RASD1*	3.8 × $10^{-61}$	*PLA2G16*	1.5 × $10^{-49}$
*CHAF1B*	3.8 × $10^{-58}$	*2F1*	1.5 × $10^{-49}$
*TRIT1*	1.7 × $10^{-56}$	*KIAA0586*	3.4 × $10^{-49}$
*PMP22*	8.1 × $10^{-55}$	*ADK*	3.7 × $10^{-49}$
*PSN1*	1.4 × $10^{-53}$	*TFRC*	4.0 × $10^{-49}$
*PLAT*	2.5 × $10^{-53}$	*CNP*	8.2 × $10^{-48}$
*BIRC5*	2.7 × $10^{-52}$	*RO1L*	3.1 × $10^{-47}$
*USP1*	2.0 × $10^{-51}$		
**Top 25 Most Significant in SKBR3 Cell Line**			
**Gene**	**Adj** ***p*****-Val**	**Gene**	**Adj** ***p*****-Val**
*CCL2*	7.7 × $10^{108}$	*HN1*	6.6 × $10^{-90}$
*F8A1*	2.7 × $10^{104}$	*C20orf24*	8.3 × $10^{-90}$
*E2F1*	1.0 × $10^{102}$	*EXO1*	1.1 × $10^{-88}$
*GINS2*	6.4 × $10^{100}$	*KIAA0101*	4.3 × $10^{-88}$
*YWHAH*	6.4 × $10^{100}$	*TFRC*	2.6 × $10^{-87}$
*PRMT6*	7.7 × $10^{100}$	*NACC2*	1.5 × $10^{-86}$
*DTL*	7.8 × $10^{-99}$	*DDIT4*	2.5 × $10^{-86}$
*GSTP1*	4.4 × $10^{-98}$	*CNOT10*	2.8 × $10^{-86}$
*BIRC5*	6.5 × $10^{-98}$	*FAHD1*	7.3 × $10^{-84}$
*DOLK*	6.5 × $10^{-98}$	*USP1*	7.3 × $10^{-84}$
*SAP30*	7.5 × $10^{-92}$	*MGST2*	1.7 × $10^{-83}$
*RPP40*	1.3 × $10^{-91}$	*BRCA1*	2.5 × $10^{-83}$
*RTN4IP1*	3.9 × $10^{-90}$		

**Table 5:** Expression of the genes and microRNAs undergoing statistically significant expression changes during the development of trastuzumab resistance in both quantitative and percentage terms.

Genes	BT474	SKBR3
all	34,756	34,756
significant p0.05	8874	13,892
significant p0.05 (%)	**25.50%**	**40.00%**
significant p0.01	4018	7529
significant p0.01 (%)	**11.60%**	**21.70%**
MicroRNAs		
all	2549	2549
significant p0.05	2540	2541
significant p0.05 (%)	**99.60%**	**99.70%**
significant p0.01	2532	2537
significant p0.01 (%)	**99.30%**	**99.50%**

**Table 6:** Top 25 most statistically significantly expressed microRNAs during the whole process of trastuzumab resistance development separately for both biological models: BT474 and SKBR3.

**Top 25 Most Significant in BT474 Cell Line**			
**microRNA**	**Adj *p*-Val**	**microRNA**	**Adj *p*-Val**
hsa-miR-574-3p	2.2 × $10^{213}$	hsa-miR-6775-3p	2.4 × $10^{177}$
hsa-miR-1207-5p	4.5 × $10^{204}$	hsa-miR-466	2.2 × $10^{174}$
hsa-miR-8485	4.3 × $10^{197}$	hsa-miR-4649-3p	2.9 × $10^{173}$
hsa-miR-6886-3p	6.5 × $10^{196}$	hsa-miR-1281	2.4 × $10^{172}$
hsa-miR-6088	3.4 × $10^{190}$	hsa-miR-6743-3p	5.9 × $10^{171}$
hsa-miR-4254	5.6 × $10^{190}$	hsa-miR-4436b-5p	5.5 × $10^{170}$
hsa-miR-4701-5p	2.2 × $10^{185}$	hsa-miR-4530	2.0 × $10^{166}$
hsa-miR-3151-3p	4.5 × $10^{184}$	hsa-miR-3162-3p	5.3 × $10^{166}$
hsa-miR-197-3p	1.7 × $10^{182}$	hsa-miR-483-3p	1.7 × $10^{164}$
hsa-miR-1825	4.5 × $10^{180}$	hsa-miR-4725-5p	1.9 × $10^{164}$
hsa-miR-6834-3p	4.5 × $10^{180}$	hsa-miR-663a	1.1 × $10^{163}$
hsa-miR-4281	5.7 × $10^{180}$	hsa-miR-6794-3p	2.9 × $10^{163}$
hsa-miR-3591-3p	1.3 × $10^{177}$		
**Top 25 Most Significant in SKBR3 Cell Line**			
**microRNA**	**Adj *p*-Val**	**microRNA**	**Adj *p*-Val**
hsa-miR-7977	1.1 × $10^{235}$	hsa-miR-5100	4.5 × $10^{163}$
hsa-miR-6826-5p	2.2 × $10^{214}$	hsa-miR-4530	7.4 × $10^{163}$
hsa-miR-7975	6.5 × $10^{203}$	hsa-miR-6869-5p	2.1 × $10^{161}$
hsa-miR-6165	1.7 × $10^{190}$	hsa-miR-6085	3.0 × $10^{161}$
hsa-miR-5739	4.6 × $10^{185}$	hsa-miR-15b-5p	1.6 × $10^{156}$
hsa-miR-574-3p	4.6 × $10^{185}$	hsa-miR-20a-5p	1.6 × $10^{156}$
hsa-let-7a-5p	3.2 × $10^{179}$	hsa-miR-574-5p	5.1 × $10^{155}$
hsa-miR-6749-5p	5.6 × $10^{176}$	hsa-miR-8485	1.7 × $10^{153}$
hsa-miR-3162-5p	8.4 × $10^{173}$	hsa-miR-197-3p	7.0 × $10^{152}$
hsa-miR-1202	1.5 × $10^{171}$	hsa-miR-6090	8.0 × $10^{149}$
hsa-miR-4284	2.6 × $10^{168}$	hsa-miR-29c-3p	1.3 × $10^{148}$
hsa-let-7e-5p	2.1 × $10^{167}$	hsa-miR-4455	4.2 × $10^{148}$
hsa-miR-24-3p	1.5 × $10^{165}$		

**Table 7:** Pair of significant genes and miRNAs where miRNA target genes were predicted by miRDB database.

miRNA.Name	Gene.Symbol	Target.Score
hsa-miR-4701-5p	*TFRC*	69
hsa-miR-8485	*E2F1*	89
hsa-miR-8485	*USP1*	51

**Table 8:** Top 10 most significant and enriched resp. Molecular Function Gene Ontology (GO) terms out of a total of 193 that have been identified for the SKBR3 cell line. A complete list of significant and enriched GO terms in Table S10.

MF GO Term Analysis for SKBR3 Cell Line					
Top 10 Most Significant MF			Top 10 Most Enriched MF		
GO Terms			GO Terms		
ID	Term	Adjusted *p*-Value	ID	Term	% Signif/All
GO:0003723	RNA binding	1.0 × $10^{-30}$	GO:0061608	nuclear import signal receptor activity	100.00
GO:0003735	structural constituent of ribosome	1.9 × $10^{-17}$	GO:0008097	5S rRNA binding	100.00
GO:0045296	cadherin binding	2.1 × $10^{-13}$	GO:0016884	carbon-nitrogen ligase activity, with glutamine as amido-N-donor	100.00
GO:0031625	ubiquitin protein ligase binding	5.5 × $10^{-13}$	GO:0140142	nucleocytoplasmic carrier activity	95.00
GO:0005524	ATP binding	2.2 × $10^{-9}$	GO:0000339	RNA cap binding	94.00
GO:0019899	enzyme binding	5.0 × $10^{-9}$	GO:0005123	death receptor binding	94.00
GO:0031492	nucleosomal DNA binding	4.6 × $10^{-8}$	GO:0031492	nucleosomal DNA binding	93.00
GO:0003697	singl-strandd DNA binding	8.4 × $10^{-8}$	GO:0030515	snoRNA binding	93.00
GO:0005525	GTP binding	2.0 × $10^{-7}$	GO:0008353	RNA polymerase II carboxy-terminal domain kinase activity	93.00
GO:0008565	protein transporter activity	2.3 × $10^{-7}$	GO:0003688	DNA replication origin binding	93.00

**Table 9:** Top 10 most significant and enriched resp. BP GO terms out of a total of 600 that have been identified for the SKBR3 cell line. Top 300 list of significant and top 20 enriched GO terms in Table S12.

BP GO Term Analysis for SKBR3 Cell Line					
Top 10 Most Significant BP			Top 10 Most Enriched BP		
GO Terms			GO Terms		
ID	Term	Adjusted *p*-Value	ID	Term	% Signif/All
GO:0051301	cell division	2.5 × $10^{-15}$	GO:0000920	cell separation after cytokinesis	100
GO:0006364	rRNA processing	2.6 × $10^{-14}$	GO:1904874	positive regulation of telomerase RNA localization to Cajal body	100
GO:0070125	mitochondrial translational elongation	2.1 × $10^{-12}$	GO:0000054	ribosomal subunit export from nucleus	100
GO:1902036	regulation of hematopoietic stem cell differentiation	3.3 × $10^{-12}$	GO:0070525	tRNA threonylcarbamoyladenosine metabolic process	100
GO:0070126	mitochondrial translational termination	5.2 × $10^{-12}$	GO:0051315	attachment of mitotic spindle microtubules to kinetochore	100
GO:0043488	regulation of mRNA stability	1.0 × $10^{-11}$	GO:0060707	trophoblast giant cell differentiation	100
GO:1901796	regulation of signal transduction by p53 class mediator	2.4 × $10^{-11}$	GO:0090646	mitochondrial tRNA processing	100
GO:0016579	protein deubiquitination	7.7 × $10^{-11}$	GO:0090151	establishment of protein localization to mitochondrial membrane	100
GO:0038061	NIK/NF-kappaB signaling	1.3 × $10^{-10}$	GO:0072425	signal transduction involved in G2 DNA damage checkpoint	100
GO:0031145	anaphase-promoting complex-dependent catabolic process	2.5 × $10^{-10}$	GO:0016024	CDP-diacylglycerol biosynthetic process	100

**Table 10:** Top 10 most significant and enriched resp. Molecular Function Gene Ontology (GO) terms out of a total of 103 that have been identified for the BT474 cell line. A complete list of significant and top 20 enriched GO terms in Table S9.

MF GO Term Analysis for BT474 Cell Line					
Top 10 Most Significant MF			Top 10 Most Enriched MF		
GO Terms			GO Terms		
ID	Term	Adjusted *p*-Value	ID	Term	% Signif/All
GO:0005515	protin binding	1.3 × $10^{-12}$	GO:0003688	DNA rplication origin binding	86.00
GO:0003697	single-stranded DNA binding	2.1 × $10^{-5}$	GO:0030983	mismatched DNA binding	86.00
GO:0003677	DNA binding	3.9 × $10^{-5}$	GO:0097617	annealing activity	85.00
GO:0003688	DNA replication origin binding	5.3 × $10^{-5}$	GO:1990825	sequence-specific mRNA binding	80.00
GO:0030983	mismatched DNA binding	5.3 × $10^{-5}$	GO:0008242	omega peptidase activity	77.00
GO:0003682	chromatin binding	7.6 × $10^{-5}$	GO:0043138	$3^{\prime }$-$5^{\prime }$ DNA helicase activity	75.00
GO:0003723	RNA binding	1.3 × $10^{-4}$	GO:0031996	thioesterase binding	73.00
GO:0097617	annealing activity	1.4 × $10^{-4}$	GO:0004303	estradiol 17-beta dehydrogenase activity activity	73.00
GO:0030331	estrogen receptor binding	9.0 × $10^{-4}$	GO:0000400	four-way junction DNA binding	69.00
GO:0048027	mRNA $5^{\prime }$-UTR binding	1.1 × $10^{-3}$	GO:0031994	insulin-like growth factor I binding	69.00

**Table 11:** Top 10 most significant and enriched resp. BP GO terms out of a total of 354 that have been identified for the BT474 cell line. A complete list of significant and enriched GO terms in Table S11.

BP GO Term Analysis for BT474 Cell Line					
Top 10 Most Significant BP			Top 10 Most Enriched BP		
GO Terms			GO Terms		
ID	Term	Adjusted *p*-Value	ID	Term	% Signif/All
GO:0051301	cell division	2.5 × $10^{-10}$	GO:0090161	Golgi ribbon formation	82.00
GO:0007062	sister chromatid cohesion	1.3 × $10^{-9}$	GO:0006744	ubiquinone biosynthetic process	79.00
GO:0000082	G1/S transition of mitotic cell cycle	2.8 × $10^{-8}$	GO:2000651	positive regulation of sodium ion transmembrane transporter activity	77.00
GO:0008283	cell proliferation	2.1 × $10^{-7}$	GO:0048715	negative regulation of oligodendrocyte differentiation	75.00
GO:0006270	DNA replication initiation	4.1 × $10^{-7}$	GO:0031573	intra-S DNA damage checkpoint	73.00
GO:0006614	SRP-dependent cotranslational protein targeting to membrane	4.3 × $10^{-7}$	GO:0000083	regulation of transcription involved in G1/S transition of mitotic cell cycle	71.00
GO:0000184	nuclear-transcribed mRNA catabolic process, nonsense-mediated decay	1.9 × $10^{-6}$	GO:0006607	NLS-bearing protein import into nucleus	71.00
GO:0019083	viral transcription	2.3 × $10^{-6}$	GO:0035461	vitamin transmembrane transport	71.00
GO:0006364	rRNA processing	1.7 × $10^{-5}$	GO:0060576	intestinal epithelial cell development	70.00
GO:0000083	regulation of transcription involved in G1/S transition of mitotic cell cycle	2.1 × $10^{-5}$	GO:0003183	mitral valve morphogenesis	70.00

**Table 12:** KEGG Pathways common for both BT474 and SKBR3 cell lines (order is based on lower adjusted *p*-value).

ID	Pathway
path:hsa04110	Cell cycle—Homo sapiens (human)
path:hsa03460	Fanconi anemia pathway—Homo sapiens (human)
path:hsa04218	Cellular senescence—Homo sapiens (human)
path:hsa04115	p53 signaling pathway—Homo sapiens (human)
path:hsa03030	DNA replication—Homo sapiens (human)
path:hsa05169	Epstein–Barr virus infection—Homo sapiens (human)
path:hsa05219	Bladder cancer—Homo sapiens (human)
path:hsa05210	Colorectal cancer—Homo sapiens (human)

**Table 13:** Statistical parameters obtained by protein–protein interaction analysis in BT474 and SKBR3 cell lines).

PPI Statistics	SKBR3	BT474
Total Genes	6588	7009
Total Unmapped Genes	128	142
Total Mapped Genes	6460	6867
Total Proteins Mapped	5871	6020
Total Unique Proteins Mapped	5871	6013
Total Edges Mapped	20,904	20,718
Total Nodes	6730	6667
Total Edges	16,421	16,301

## Data Availability

The data presented in this study are available in Supplementary Files such as Supplementary tables in Spreadsheet and Supplementary figures in Documents.

## Supplementary Materials

The following supporting information can be downloaded at: https://www.mdpi.com/article/10.3390/cimb46030171/s1.
