# Supplementary material for: Unveiling the Molecular Mechanism of Trastuzumab Resistance in SKBR3 and BT474 Cell Lines for HER2 Positive Breast Cancer
Source: Curr Issues Mol Biol. 2024 Mar 21;46(3):2713–40. doi: 10.3390/cimb46030171 (PMC10969458; doi:10.3390/cimb46030171)
Supplement: Supplementary file 1 [file cimb-46-00171-s001.zip › Supplementary_data_fig_revised.pdf]

Supplementary Materials: Figures

Figure S1. Concentration and RNA integrity evaluation for representative number of samples using Agilent Bioanalyzer. Sample ID description: BT474, SKBR3 - RNA extracted for particular cell line; T1,T2,T3,T4,T5,T7, control - RNA extracted for particular time point. (A) Visualisation of electrophoresis. (B) Histograms presenting intensity of the signal (amount of nucleic acid) as a function of fragment size. Both (A) and (B) present samples at high RNA integrity level.

(A)

(B)

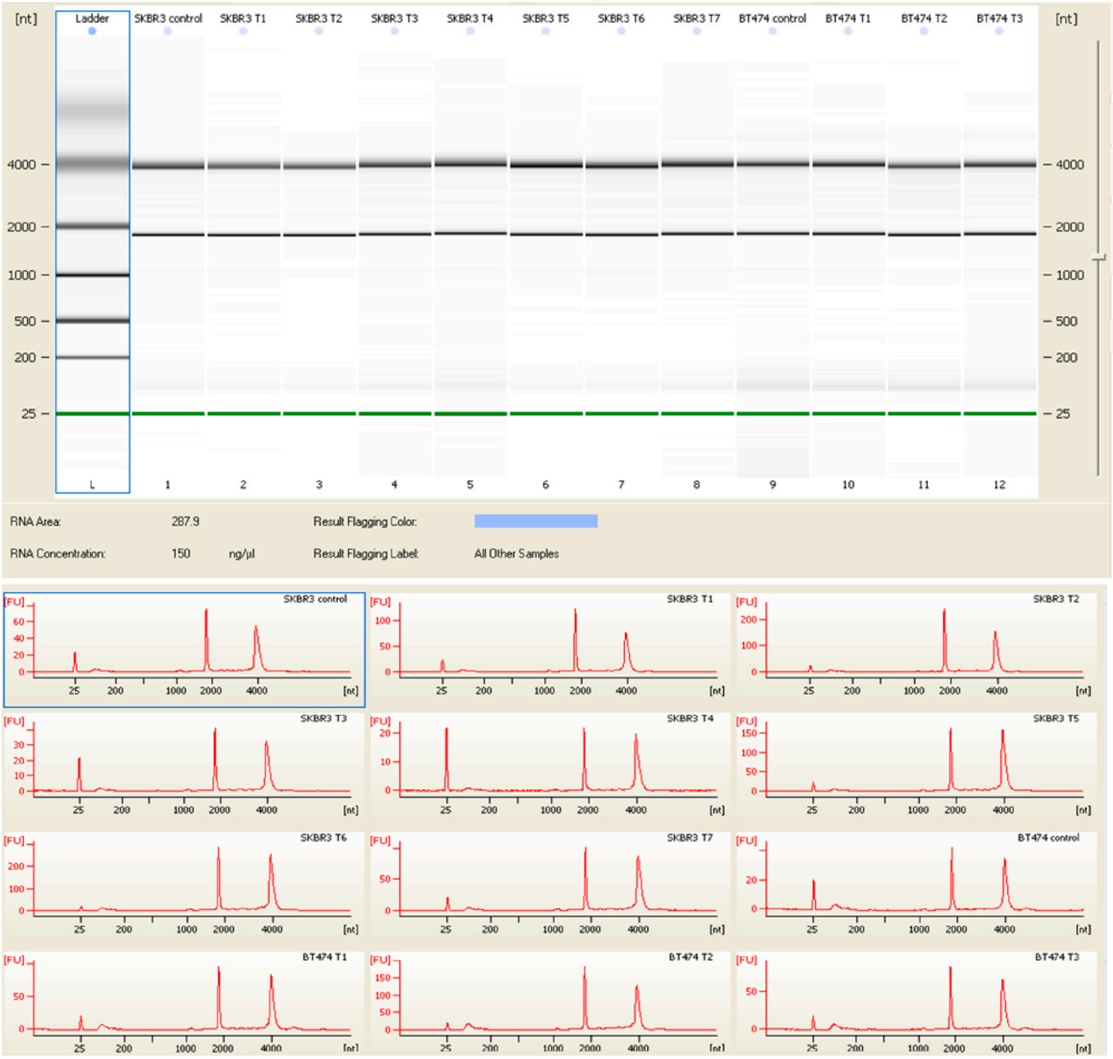

Figure S2. Volcano plots of global gene expression variations in the BT474 (left) and SKBR3 (right) cell lines. The graphs depict statistical significance (p value) vs. gene expression fold change.

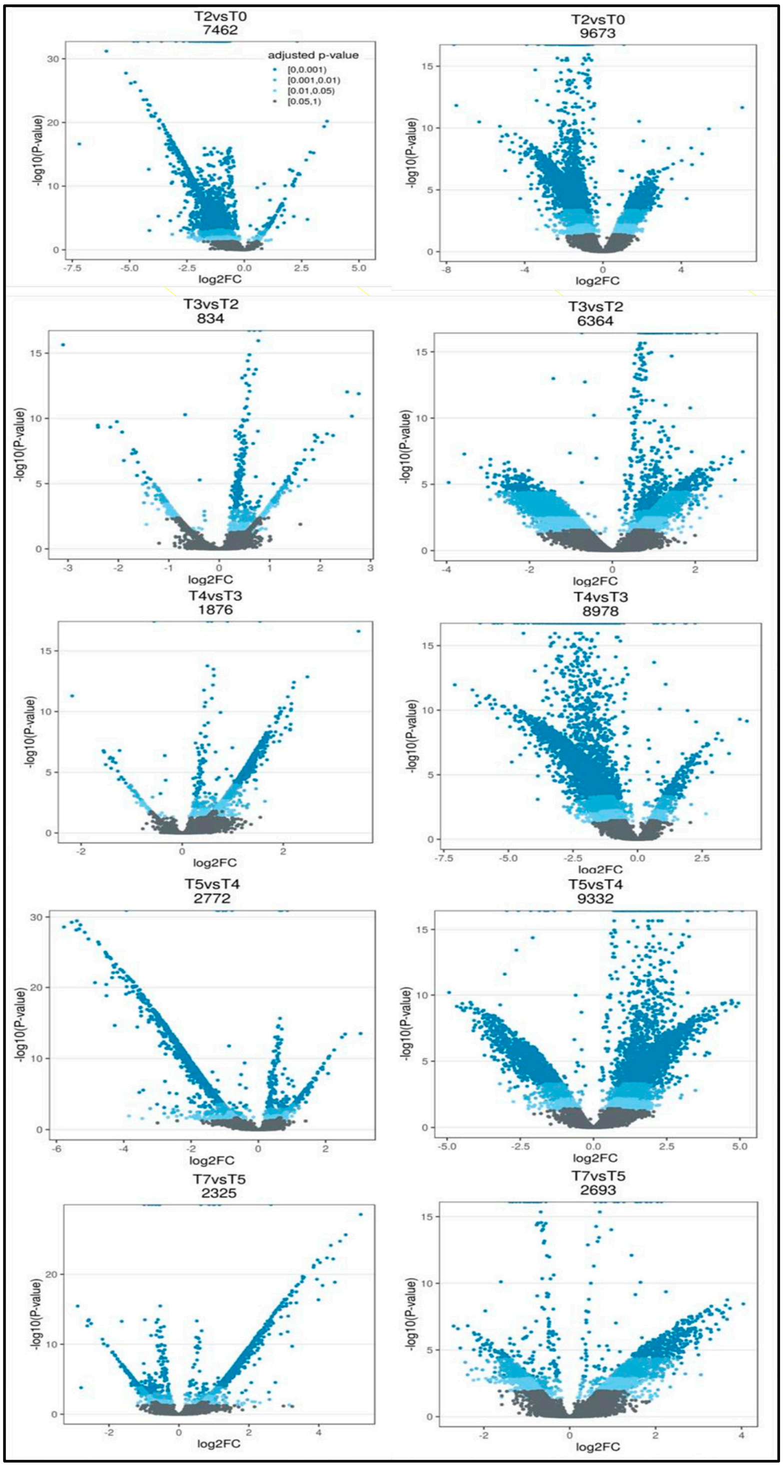

Figure S3. Density plots from the 20 most significant MF GO terms identified in the BT474 cell line. These plots illustrate how genes are distributed in terms of their p-values. Part A of the analysis focused on GO terms that exhibit a distribution pattern similar to random distribution.

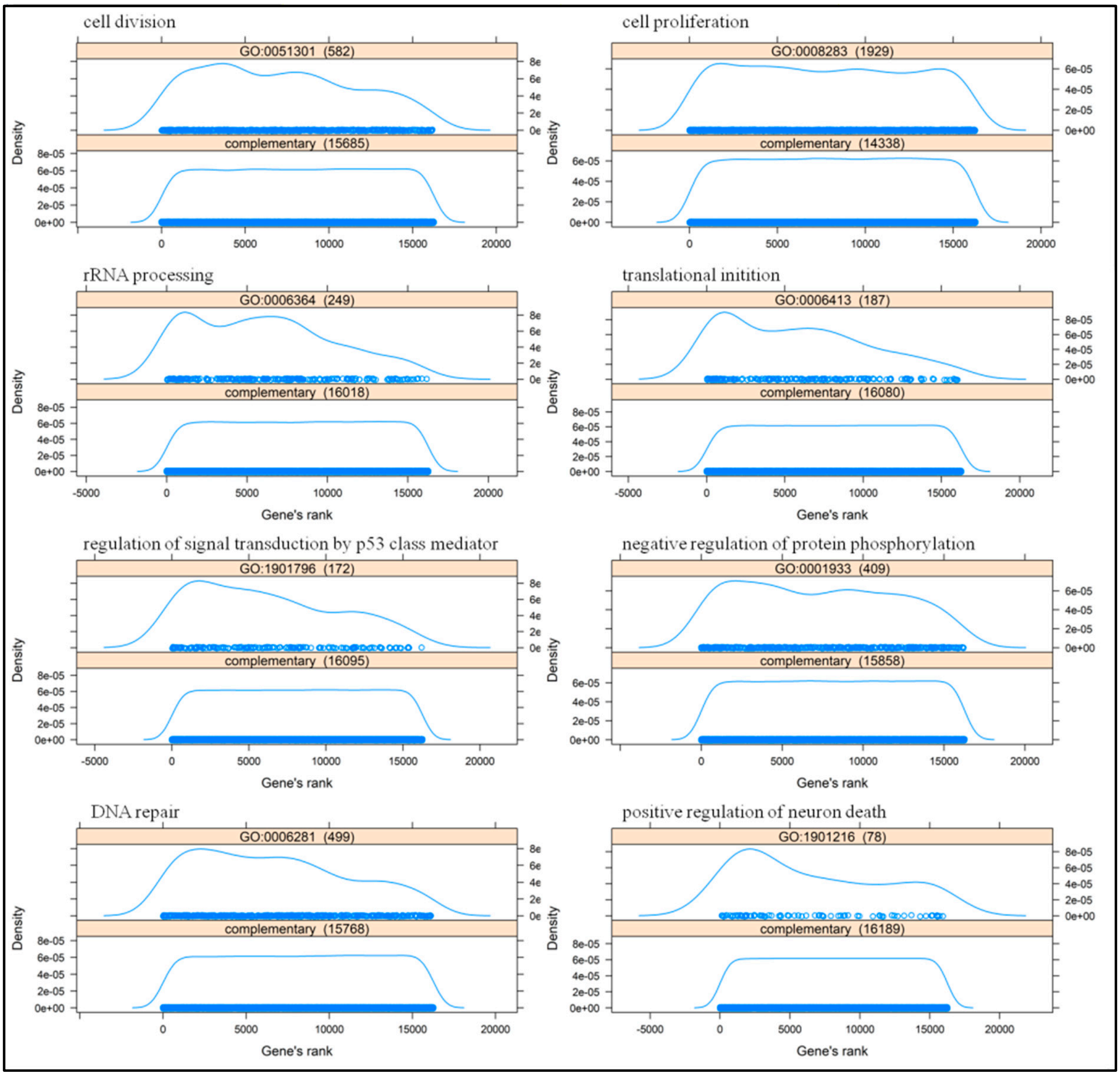

Figure S4. Density plots for the top 20 most statistically significant MF GO terms identified in the BT474 cell line. These plots illustrate the distribution of genes, ranked by their p-values. Part B of the analysis focuses on GO terms that exhibit an asymmetric distribution, enriched with genes with lower p-values, indicating higher statistical significance.

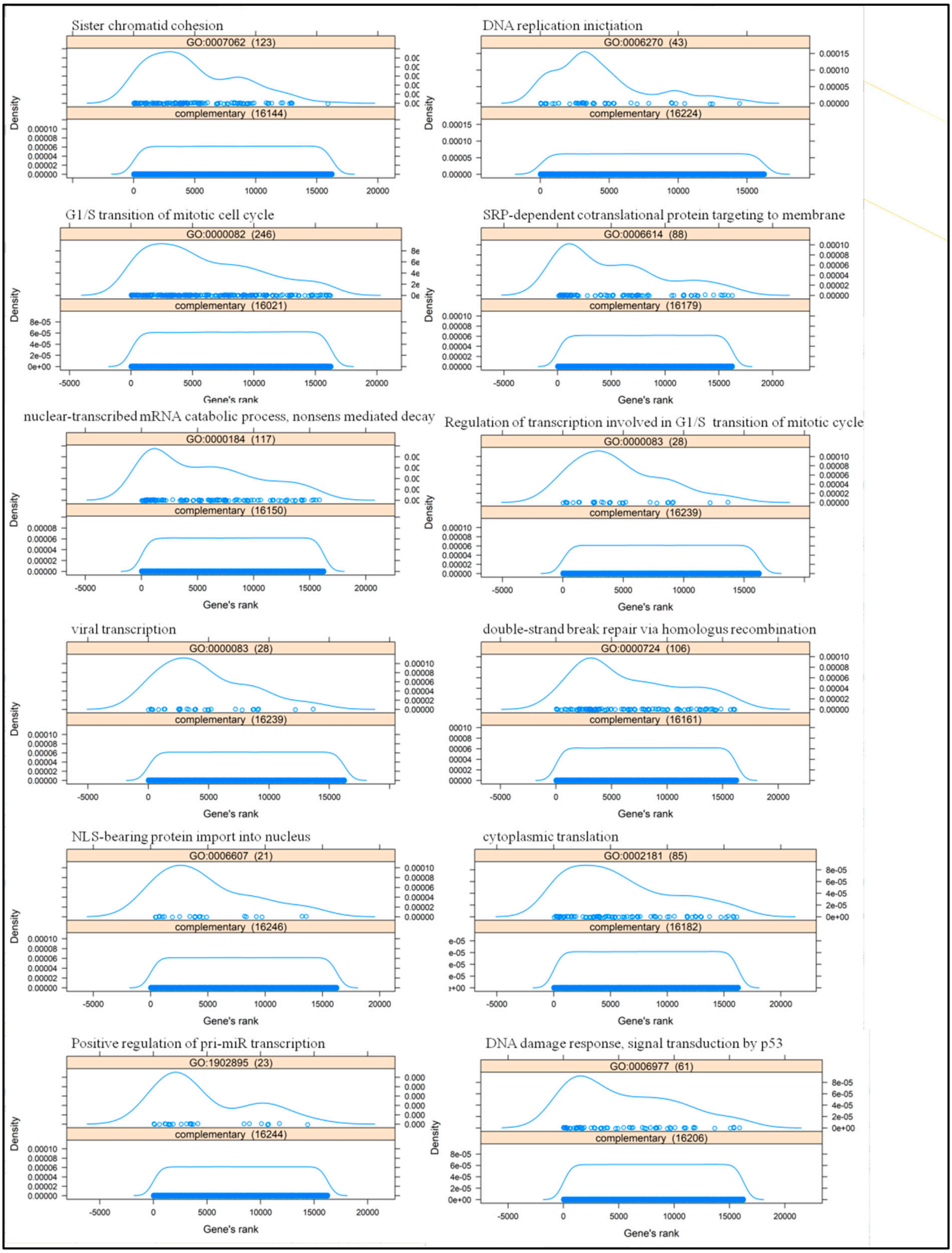

Figure S5. Density plots from the 20 most significant MF GO terms identified in the SKBR3 cell line. These plots illustrate how genes are distributed in terms of their p-values. Part A of the analysis focused on GO terms that exhibit a distribution pattern similar to random distribution.

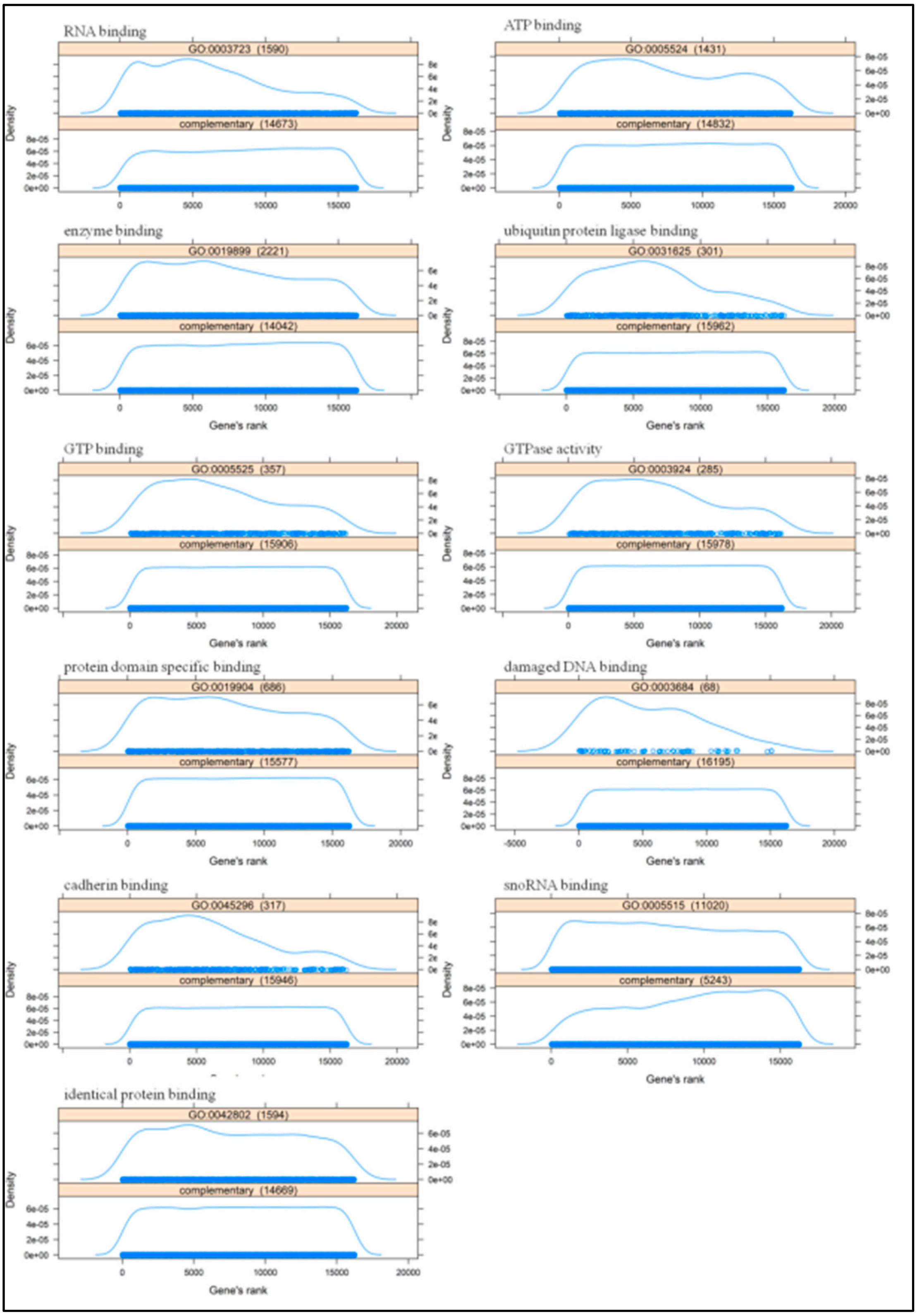

Figure S6. Density plots for the top 20 most statistically significant MF GO terms identified in the SKBR3 cell line. These plots illustrate the distribution of genes, ranked by their p-values. Part B of the analysis focuses on GO terms that exhibit an asymmetric distribution, enriched with genes with lower p-values, indicating higher statistical significance.

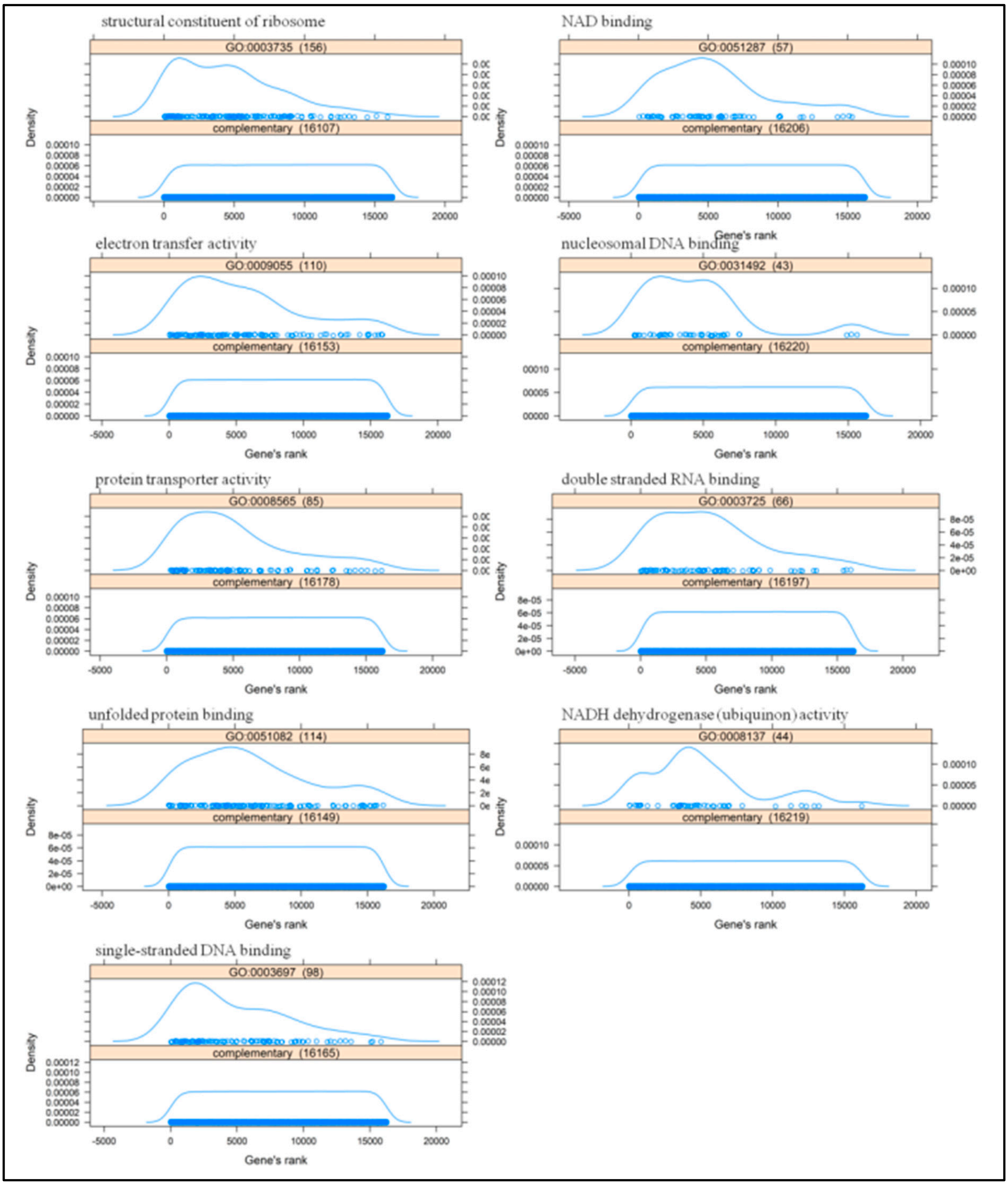

Figure S7. Density plots for top 20 most significant BP GO terms found for BT474 cell line. The plots present the distribution of genes ranked based on p-value. Part A - GO terms presenting distribution similar to random distribution.

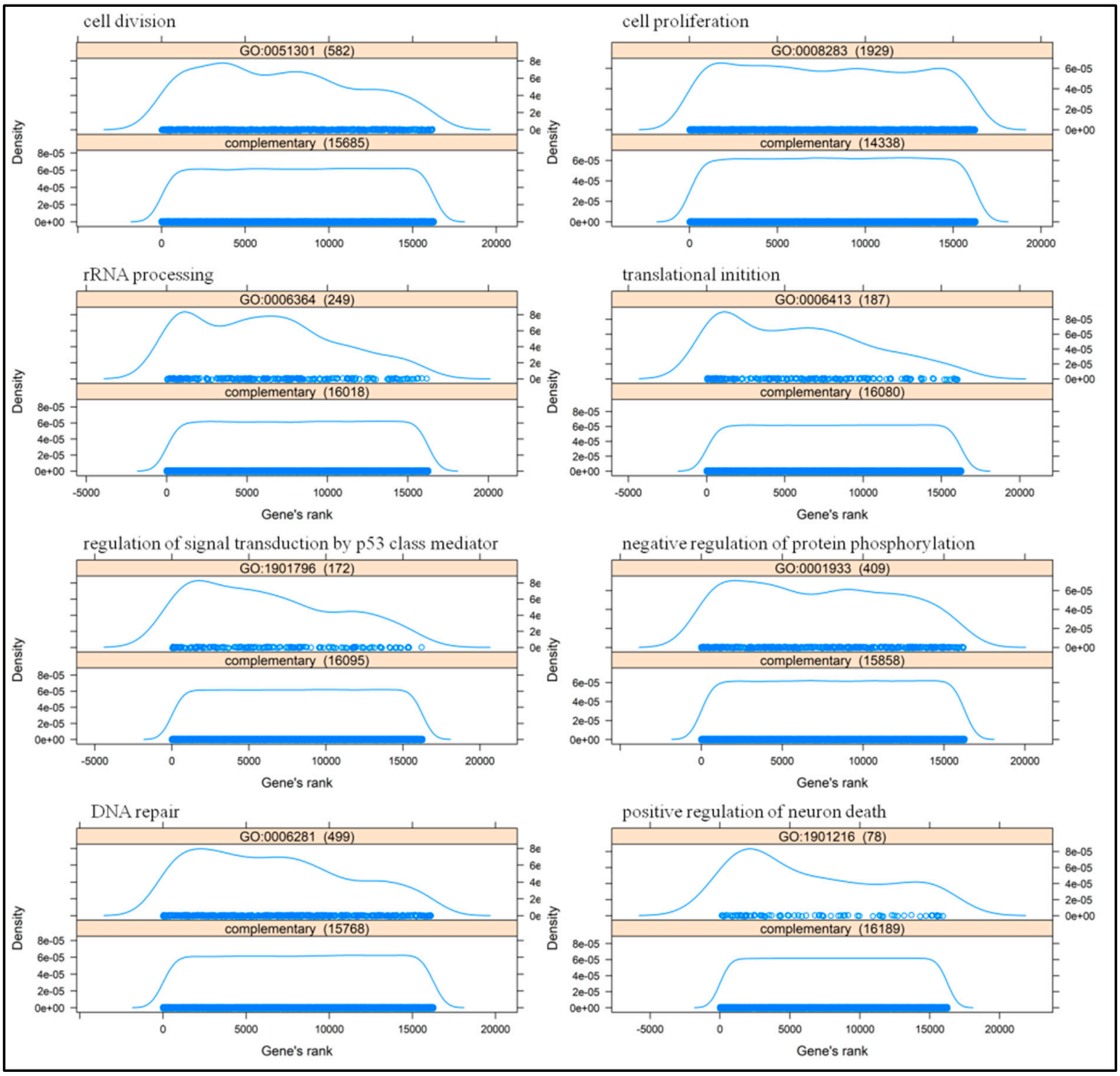

Figure S8. Density plots for top 20 most significant BP GO terms found for BT474 cell line. The plots present the distribution of genes ranked based on p-value. Part B - GO terms presenting asymetric distribution, enriched in genes characterized by lower p-value (statistically more significant)

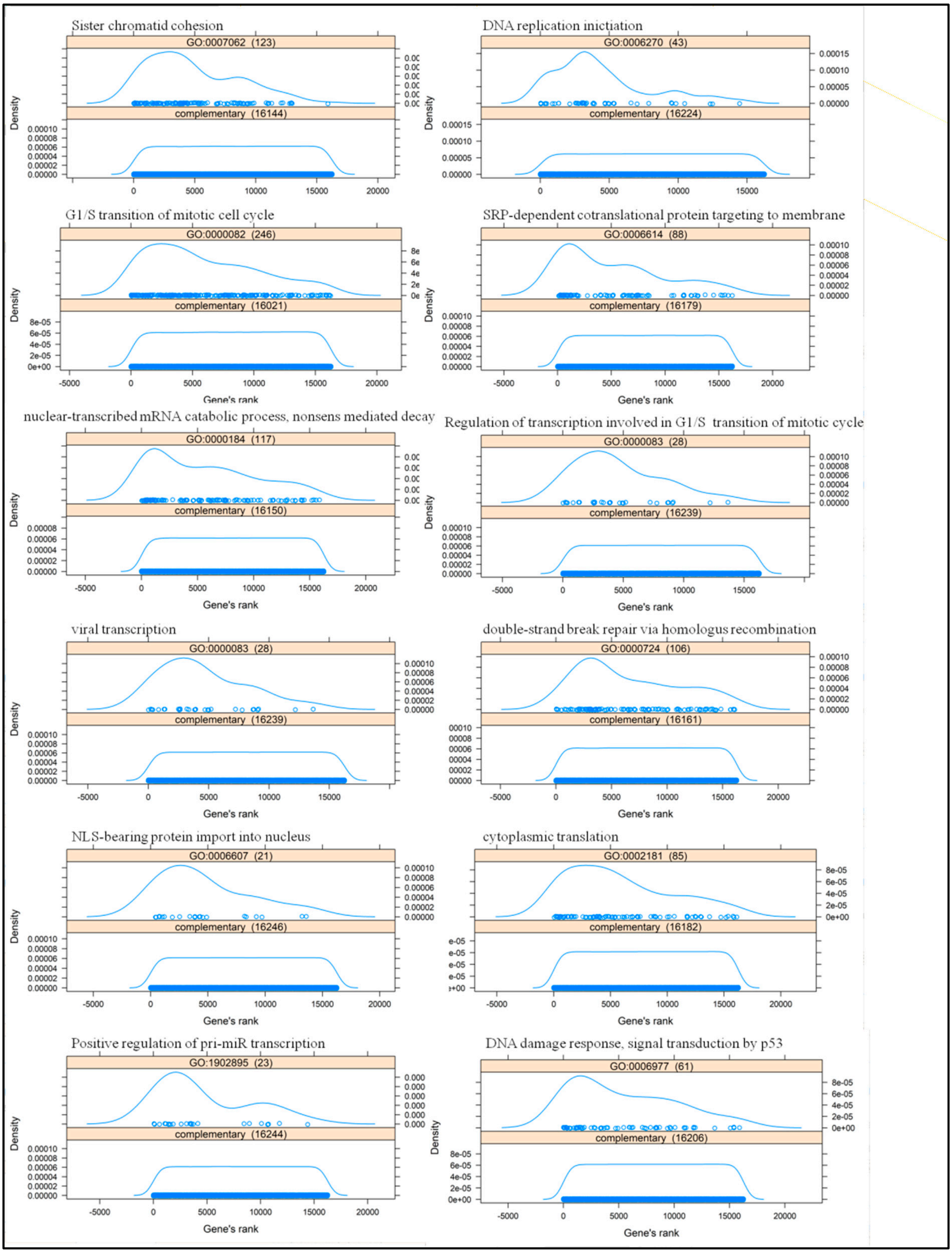

Figure S9. Density plots from the 20 most significant BP GO terms identified in the SKBR3 cell line. These plots illustrate how genes are distributed in terms of their p-values. Part A of the analysis focused on GO terms that exhibit a distribution pattern similar to random distribution.

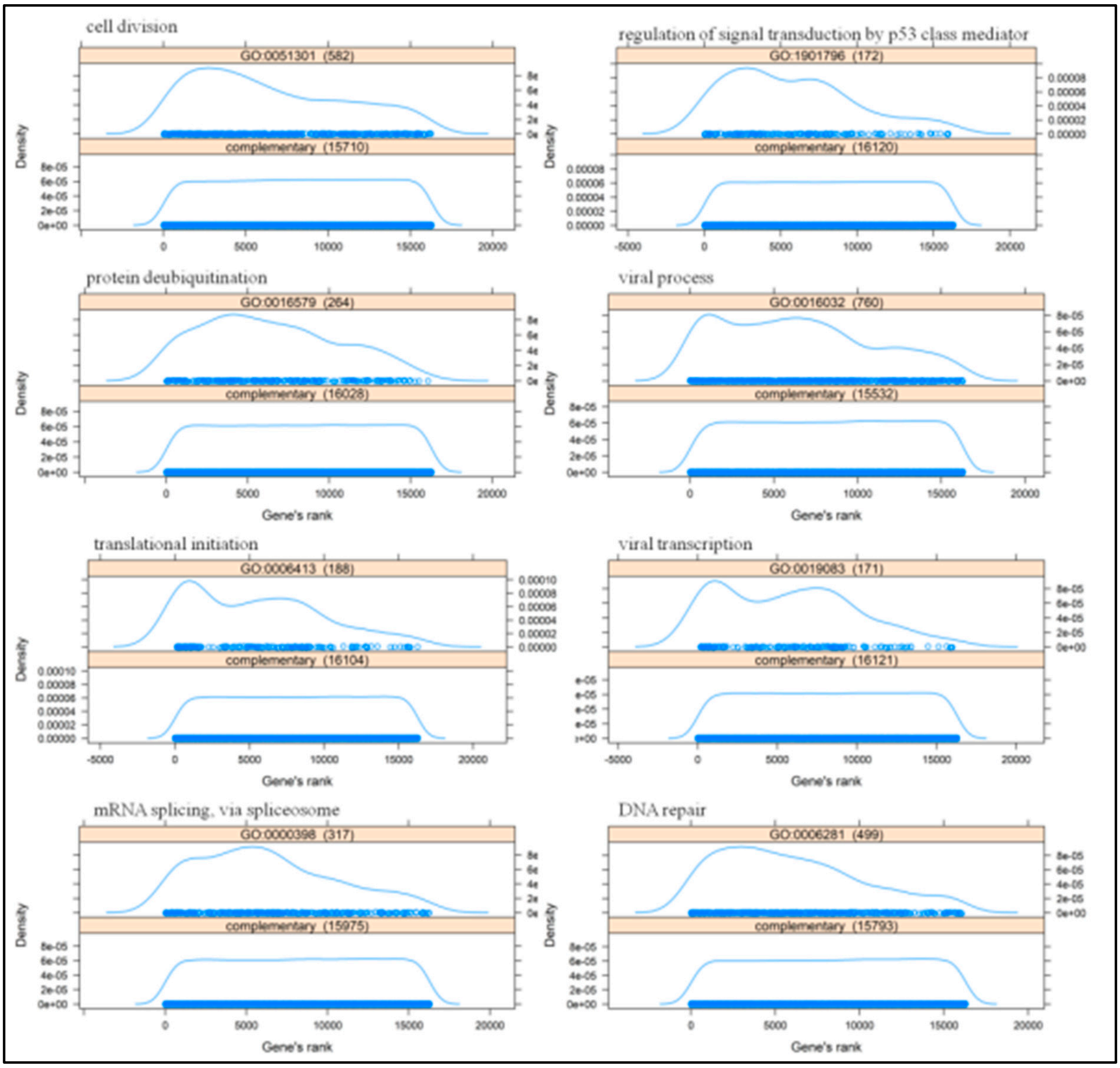

Figure S10. Density plots for the top 20 most statistically significant BP GO terms identified in the SKBR3 cell line. These plots illustrate the distribution of genes, ranked by their p-values. Part B of the analysis focuses on GO terms that exhibit an asymmetric distribution, enriched with genes with lower p-values, indicating higher statistical significance.

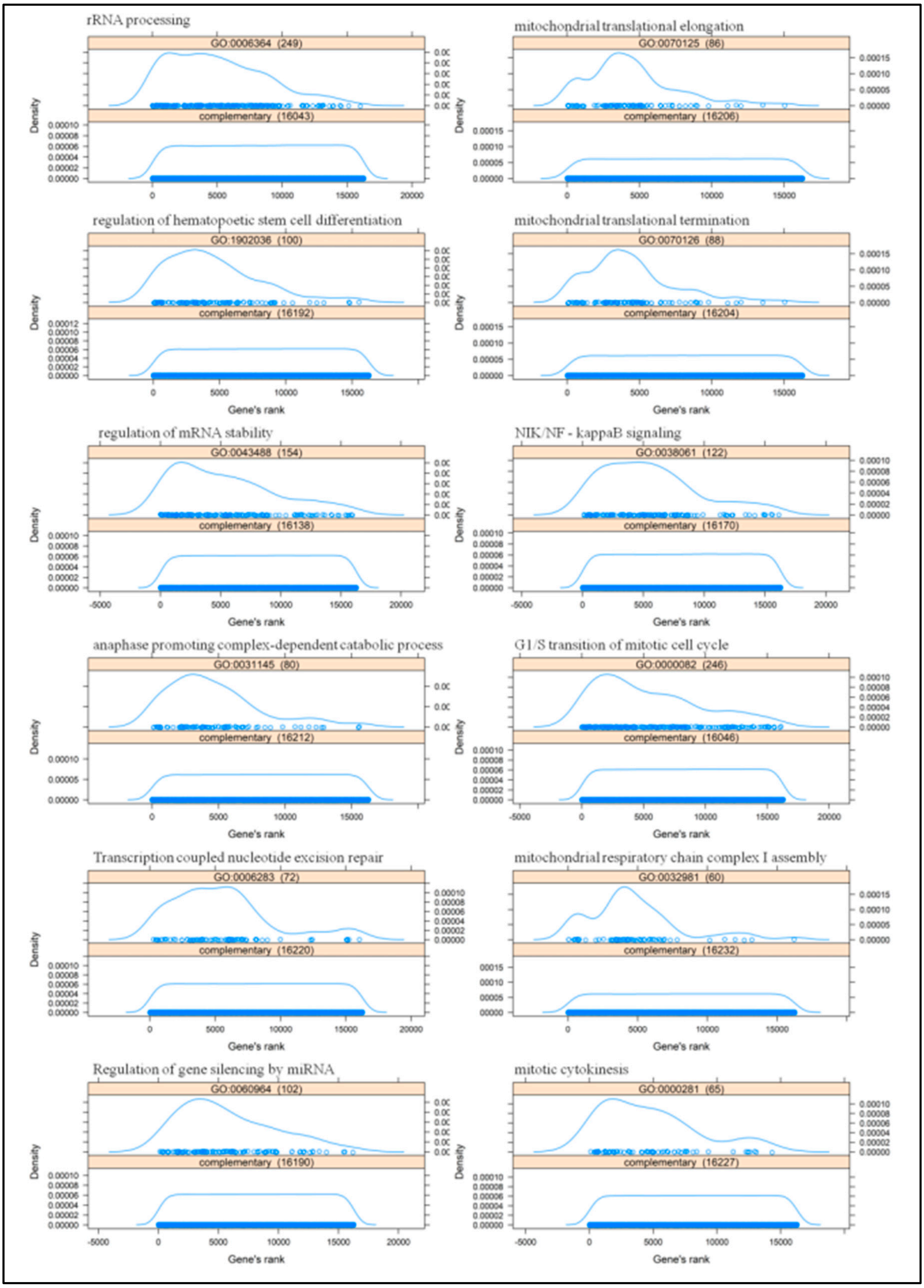

Figure S11. PPI sub-network focused on the E2F1 gene, incorporating the top 25 most significant genes in the BT474 cell line and genes associated with trastuzumab resistance development.

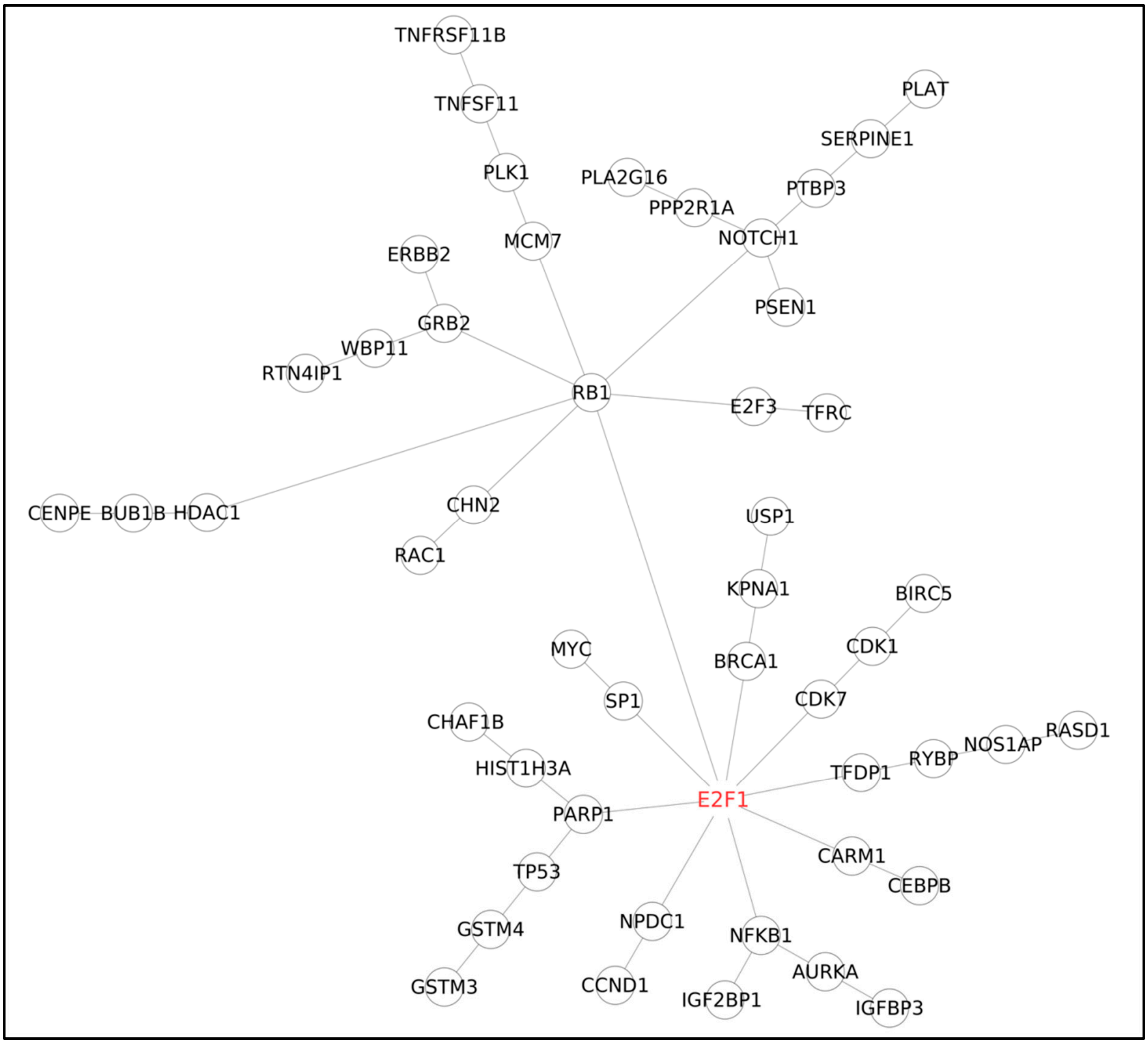

Figure S12. PPI sub-network focused on the E2F1 gene, incorporating the top 25 most significant genes in the SKBR3 cell line and genes associated with trastuzumab resistance development.

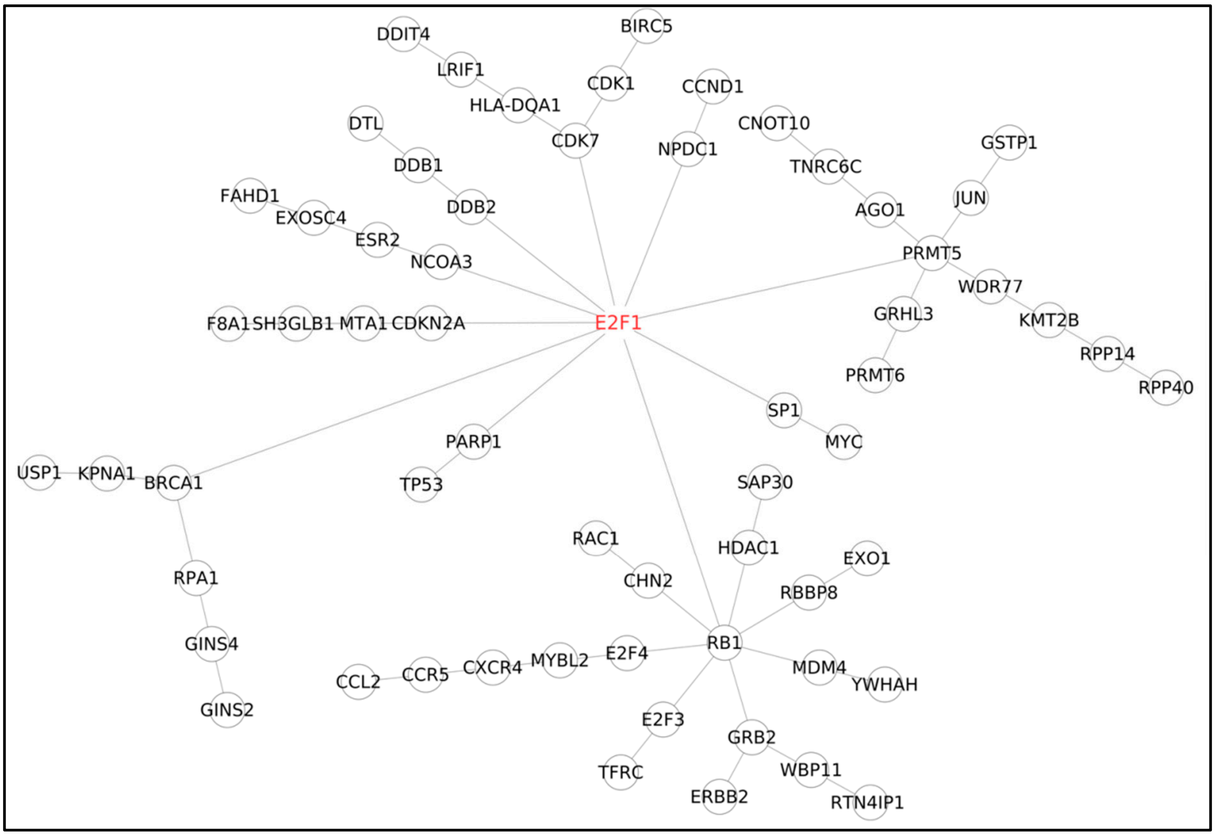

Figure S13. PPI sub-network focused on the TFRC gene, incorporating the top 25 most significant genes in the BT474 cell line and genes associated with trastuzumab resistance development.

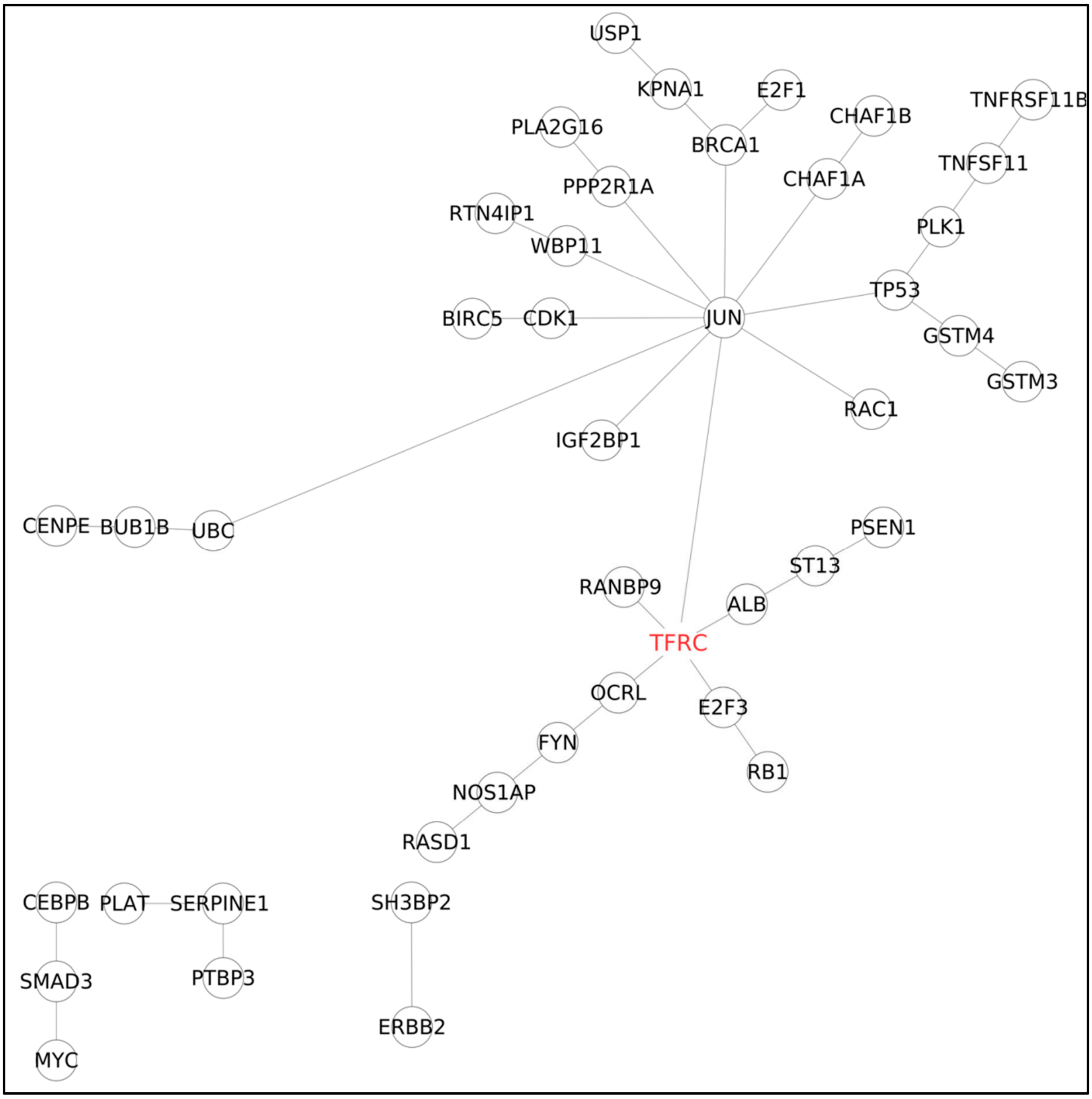

Figure S14. PPI sub-network focused on the TFRC gene, incorporating the top 25 most significant genes in the SKBR3 cell line and genes associated with trastuzumab resistance development.

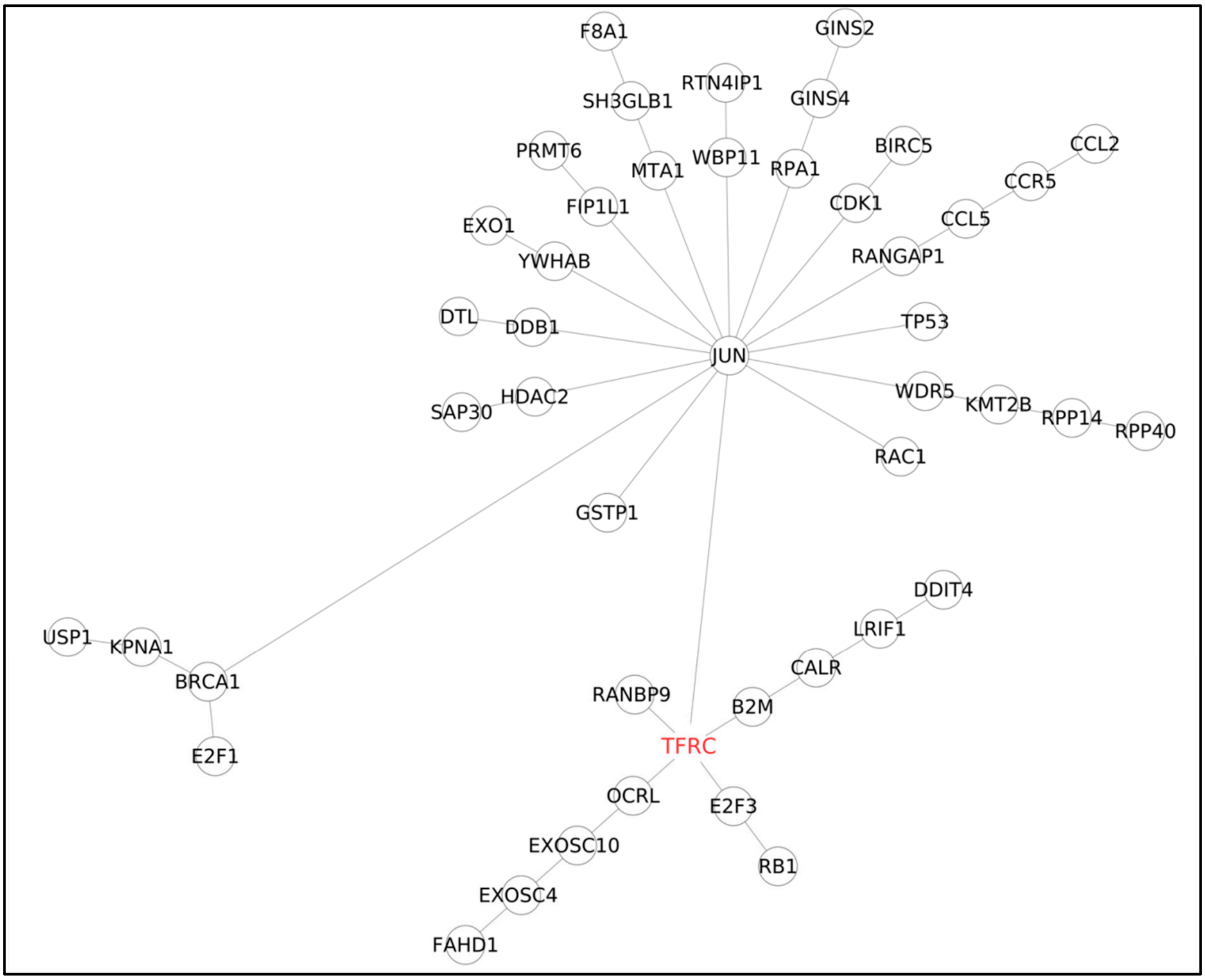

Figure S15. PPI sub-network focused on the USP1 gene, incorporating the top 25 most significant genes in the BT474 cell line and genes associated with trastuzumab resistance development.

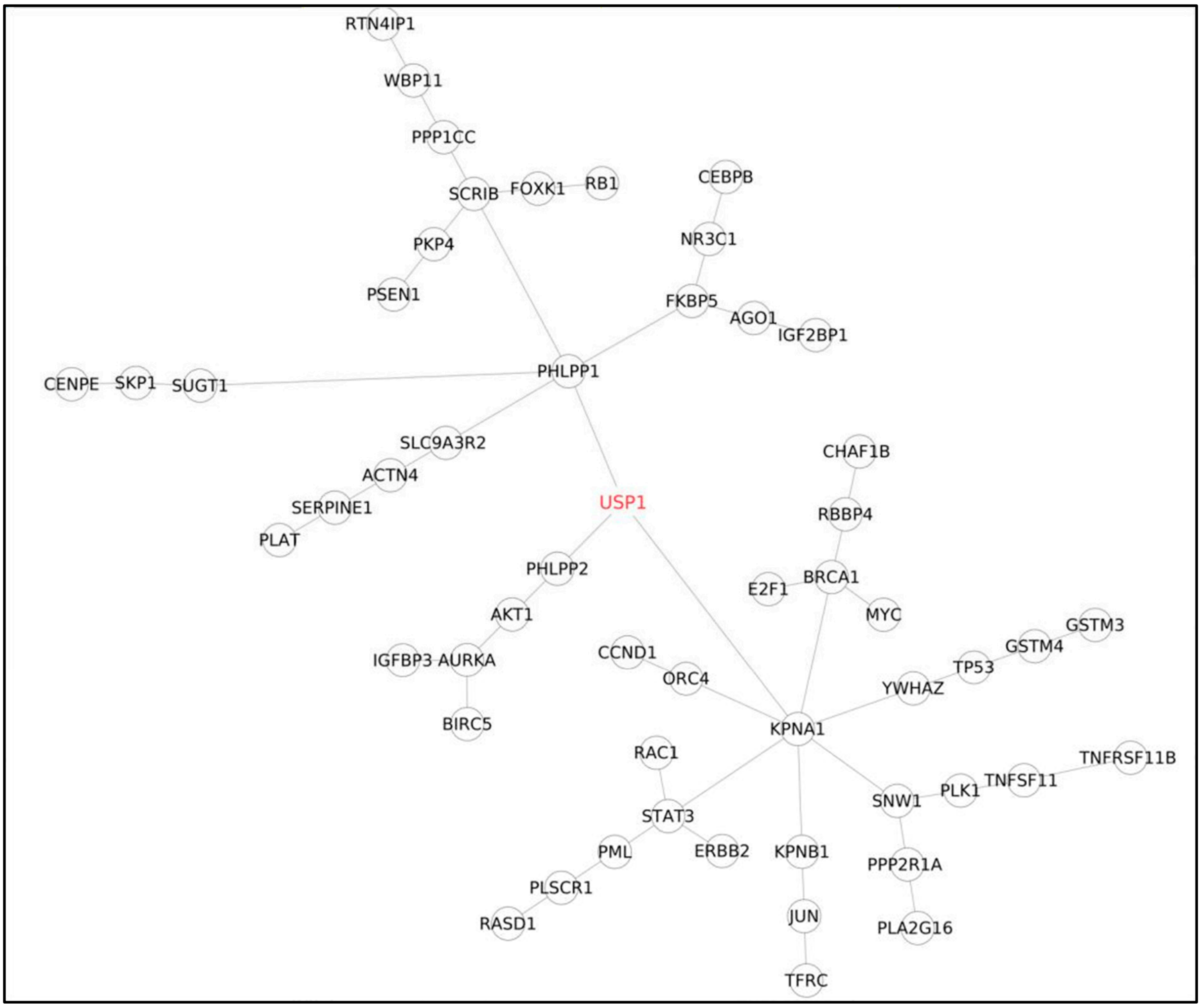

Figure S16. PPI sub-network focused on the USP1 gene, incorporating the top 25 most significant genes in the SKBR3 cell line and genes associated with trastuzumab resistance development.

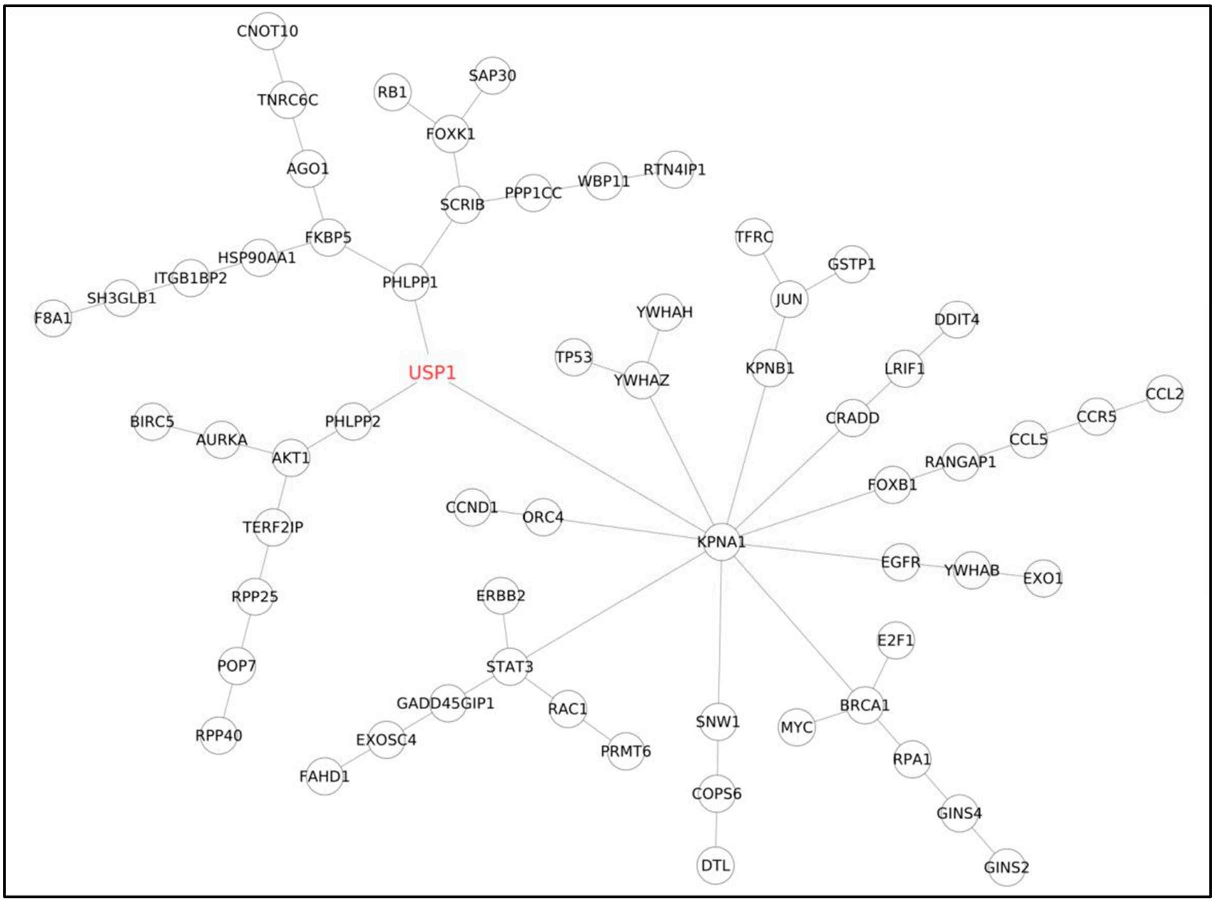

Figure S17. Box plot and line plot of distribution of log2 fold change (log2FC) values of miRNA-target-gene pair, at their average and respective time points

BT474 cell line

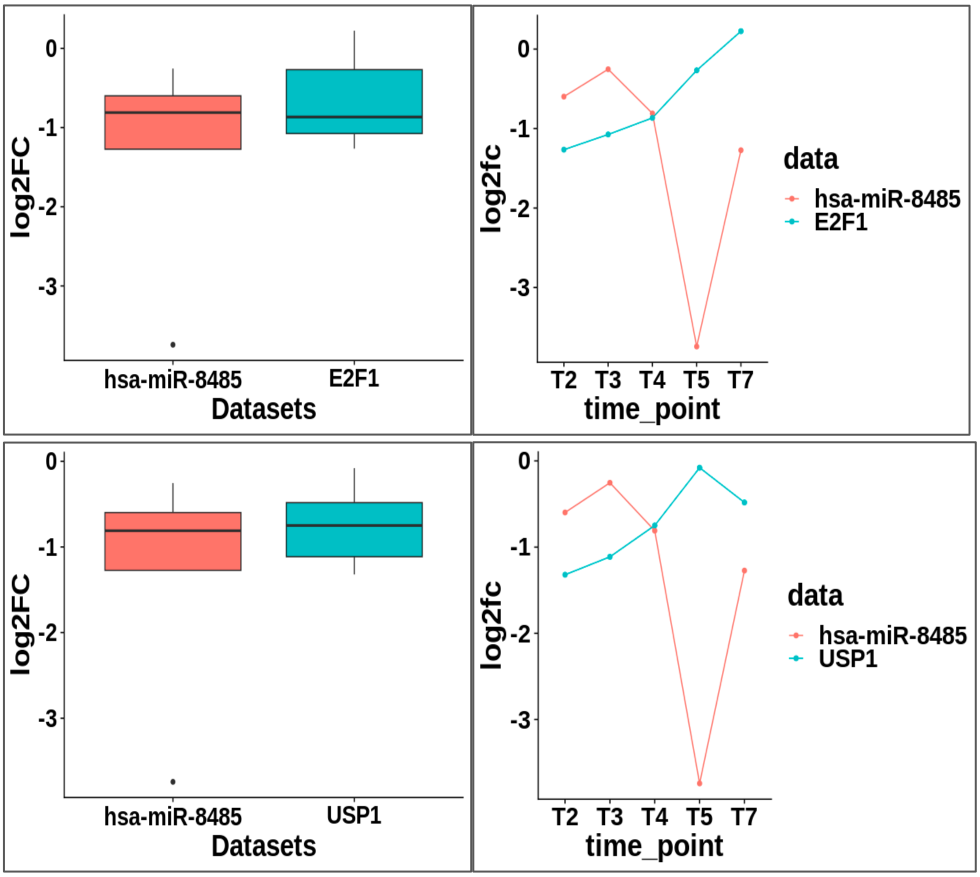

SKBR3 cell line

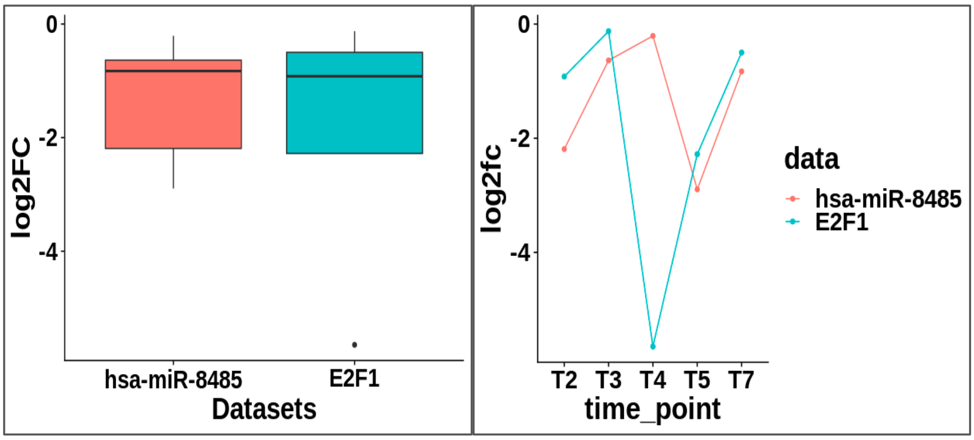

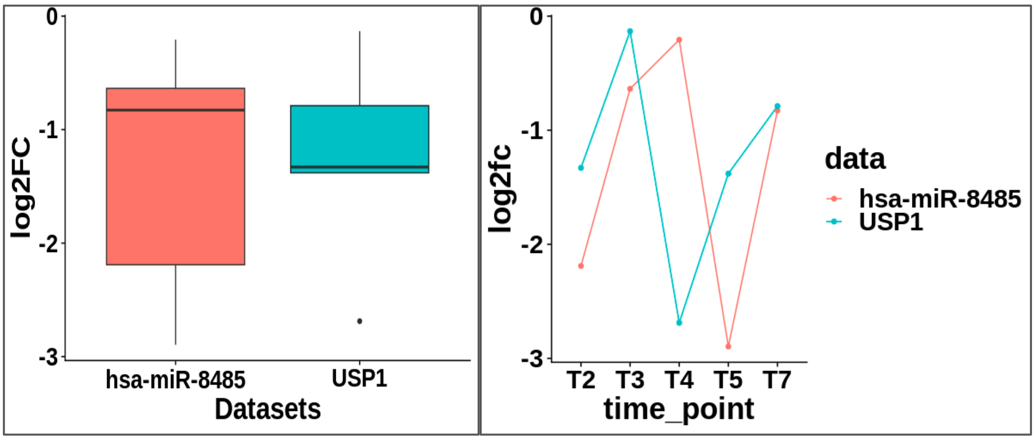

log2FC
0
-1
-2
-3
hsa-miR-8485
USP1
Datasets
log2fc
0
-1
-2
-3
T2
T3
T4
T5
T7
time_point
data
hsa-miR-8485
USP1
